# Extracellular vesicle RNAs reflect placenta dysfunction and are a biomarker source for preterm labour

**DOI:** 10.1111/jcmm.13570

**Published:** 2018-03-08

**Authors:** Shannon Fallen, David Baxter, Xiaogang Wu, Taek‐Kyun Kim, Oksana Shynlova, Min Young Lee, Kelsey Scherler, Stephen Lye, Leroy Hood, Kai Wang

**Affiliations:** ^1^ Institute for Systems Biology Seattle WA USA; ^2^ Lunenfeld‐Tanenbaum Research Institute Mount Sinai Hospital Toronto ON Canada; ^3^ Department of Obstetrics & Gynecology University of Toronto Toronto ON Canada; ^4^ Department of Physiology University of Toronto Toronto ON Canada

**Keywords:** exosome, extracellular vesicle, microRNA, next‐generation sequencing, preterm birth, preterm labour

## Abstract

Preterm birth (PTB) can lead to lifelong complications and challenges. Identifying and monitoring molecular signals in easily accessible biological samples that can diagnose or predict the risk of preterm labour (PTL) in pregnant women will reduce or prevent PTBs. A number of studies identified putative biomarkers for PTL including protein, miRNA and hormones from various body fluids. However, biomarkers identified from these studies usually lack consistency and reproducibility. Extracellular vesicles (EVs) in circulation have gained significant interest in recent years as these vesicles may be involved in cell‐cell communication. We have used an improved small RNA library construction protocol and a newly developed size exclusion chromatography (SEC)‐based EV purification method to gain a comprehensive view of circulating RNA in plasma and its distribution by analysing RNAs in whole plasma and EV‐associated and EV‐depleted plasma. We identified a number of miRNAs in EVs that can be used as biomarkers for PTL, and these miRNAs may reflect the pathological changes of the placenta during the development of PTL. To our knowledge, this is the first study to report a comprehensive picture of circulating RNA, including RNA in whole plasma, EV and EV‐depleted plasma, in PTL and reveal the usefulness of EV‐associated RNAs in disease diagnosis.

## 1 INTRODUCTION

Preterm birth (PTB) is childbirth occurring at <37 completed weeks of gestation.^1^ PTB is the second largest direct cause of death in children younger than 5 years, and complications associated with PTB are estimated to be responsible for 35% of the world's 3.1 million annual neonatal deaths.^2^ Premature babies have higher rates of cerebral palsy, sensory deficits, learning disabilities and respiratory illnesses that extend into adulthood.^1^ This increased lifelong morbidity results in high economic and social costs to families and communities.^1^, ^2^ Identifying and monitoring molecular signals in easily accessible body fluids that can diagnose or predict the risk of preterm labour (PTL) in pregnant women will reduce or prevent PTBs.

Protein has been the major player of diagnostic markers used in the clinic. For example, prostate‐specific antigen (PSA) is measured for prostate cancer progression^3^ and alpha foetal protein for liver‐related conditions.^4^ In recent years, cell‐free circulating nucleic acids, especially circulating microRNA (miRNA), have garnered much attention for their potential as a disease biomarker. MiRNAs are evolutionary conserved, small non‐coding RNAs ranging in size from 19 to 24 nucleotides. They regulate various biological activities by modulating the cellular transcriptome and proteome.^5^, ^6^ Besides their regulatory roles in the cell, miRNAs can also be detected in various body fluids. These cell‐free circulating miRNAs are either bound to RNA‐binding proteins, such as NPM1 or Ago2,^7^ or lipoproteins, such as HDL or LDL,^8^ or encapsulated into extracellular vesicles (EVs) to escape RNase degradation.^6^, ^9^ Some circulating miRNAs are already used as markers for disease diagnosis or prognosis. For example, the circulating miR‐122 level is closely associated with different liver diseases 10 and miR‐208 level is associated with heart conditions.^11^, ^12^ It has been suggested that at least some of the EVs, including exosomes, are involved in cell‐cell communication.^13^ Therefore, characterizing the molecular content and studying the function of EVs have been of great interest in recent years.

A number of studies report putative biomarkers for PTL including protein, miRNA and hormones from various body fluids such as serum and plasma,^14^, ^15^ cervical vaginal fluid,^15^ saliva and amniotic fluids.^15^ They can largely be grouped into three main categories: inflammatory‐related molecules, placenta or foetal‐derived molecules and stress‐related molecules. For example, several inflammation‐related proteins, including C‐reactive protein and cytokines, including IL6, IL8 and IL10, show PTL‐associated concentration changes.^16^, ^17^, ^18^, ^19^ However, biomarkers identified from these studies lack consistency, especially for cell‐free miRNA‐based biomarkers. For example, multiple studies report changes of specific miRNA concentrations in serum or plasma of PTL patients, but the results are inconsistent or even contradictory among studies.^20^, ^21^, ^22^, ^23^, ^24^ Elovitz et al, using microarray, concluded that PTL has very little effect on serum‐derived miRNA; however, Gray et al, using the nanostring platform, identified several miRNAs that can be used to predict the development of PTL.^23^, ^24^ The major causes of inconsistency are different types of sample used (serum vs. plasma), low RNA concentration in samples and lack of robust measurement technology. In the past few years, next‐generation sequencing (NGS) has become the major platform for miRNA analysis, especially for body fluid samples. Yet studies have shown significant sequence bias among different small RNA library preparation protocols.^25^, ^26^ We adapted a small RNA library construction protocol using adapters with 4 degenerated nucleotides at miRNA‐adapter ligation ends to reduce ligation‐associated sequence bias. In addition, ultracentrifugation has been the method of choice for EV purification but the high centrifugation force may alter the natural state and content of EVs. Furthermore, the method is low throughput and requires large sample volume. We tested a newly developed size exclusion chromatography (SEC)‐based EV purification protocol that provides higher throughput and cleaner EVs compared to other methods.^27^, ^28^ We are using these improved approaches to gain more reliable profiles of circulating RNA in body fluid as well as its associated EVs and EV‐depleted plasma to explore the possibility of using circulating miRNAs, specifically those encapsulated in EVs, as a non‐invasive biomarker for PTL. To our knowledge, this is the first comprehensive characterization of circulating RNA encompassing whole plasma, EV and EV‐depleted plasma in PTL.

## 2 MATERIALS AND METHODS

### 2.1 Ethics statement

This study was approved by the Research Ethics Board of Mount Sinai Hospital, Toronto, Canada (#04‐0024‐E), and was conducted according to the principles of Declaration of Helsinki. All patients provided written informed consent to participate in the Global Alliance to Prevent Prematurity and Stillbirth (GAPPS) study.

### 2.2 Patient and sample information

Blood samples were collected from women who had PTL and gestational age‐matched healthy pregnant women. Inclusion criteria for the study were presentation between 24 and 34 weeks of gestation with uterine contractions and cervical dilation <4 cm. Women who had antepartum haemorrhage, clinical chorioamnionitis, foetal anomaly, preeclampsia, intrauterine growth restriction, diabetes mellitus or gestational diabetes were excluded. The women formed part of the Ontario Birth Study cohort, at Sinai Health System, Toronto, Canada. For all preterm labouring patients, peripheral blood samples were collected prospectively at the point of hospital admission (PTL group, N = 20), while blood samples from healthy control women matched with respect to gestational age and other variables were collected during the regular antenatal visit (TL group, N = 47). All controls delivered at full term (≥37 gestational weeks).

### 2.3 Sample collection, extracellular vesicle isolation and electron microscopy

Blood samples were collected from women who participated in the GAPPS study into EDTA‐treated blood collection tubes, and the plasma was prepared according to standard protocol. Briefly, whole‐blood samples were centrifuged for 15 minutes at 2000× *g* at 4°C. The resulting supernatant is designated plasma. The plasma was transferred to clean polypropylene tubes in 100 μL aliquots and frozen at −80°C. Prior to exosome isolation or RNA extraction, plasma was spun at 10 000× *g* for 15 minutes at 4°C to remove platelets and large particles. EVs were isolated from 100 μL of plasma from a total of 22 selected samples, 11 from each group (Table S1), using size exclusion chromatography (SEC) columns (iZON qEV, Cambridge, MA) with degassed 1X PBS (pH 7.2; Gibco, Grand Island, NY). The detailed description for EV and EV‐depleted plasma preparation is in Supporting information. To confirm the purification of EVs from samples, the samples were examined with transmission electron microscopy at the Fred Hutchinson Cancer Research Center following the method as previously described.^29^ The characterization of EV is detailed in the Supporting information.

### 2.4 Isolation of RNA

RNA was isolated from all 67 plasma samples, the 22 EV samples and 22 corresponding EV‐depleted plasmas using miRNeasy Micro Kit (Qiagen, Germantown, MD). The quality and quantity of the RNA were evaluated with the Agilent 2100 Bioanalyser (Santa Clara, CA) and NanoDrop 1000 spectrophotometer (Thermo Scientific, Wilmington, DE).

### 2.5 Small RNA sequencing library construction

Small RNA sequencing libraries were generated using a modified small RNAseq protocol (available at http://exrna.org/resources/protocols/). Key elements of the protocol include the addition of 4 random nucleotides at the appropriate end of the adapters to reduce ligation‐associated sequence bias, the use of higher adapter concentrations and the increased amounts of polyethylene glycol in the ligation steps. In addition, to reduce the adapter‐dimer, an initial size selection is performed after 4 cycles of library amplification followed by a second size selection after 11‐16 cycles of amplification of library from the first size selection step. Individual library concentrations were measured using the NEBNext Library Quant Kit (New England Biolabs, Ipswich, MA) and adjusted to a final pooled concentration of 2 nmol/L and sequenced using the NEXTseq DNA sequencer (Illumina, San Diego, CA).

### 2.6 RNASeq data analysis

The raw sequence data have been deposited in NCBI's Gene Expression Omnibus 30 and are accessible through GEO Series accession number GSE106224 (https://www.ncbi.nlm.nih.gov/geo/query/acc.cgi?acc=GSE106224). The results were analysed using an in‐house small RNA analysis pipeline, sRNAnalyser (available at http://srnanalyzer.systemsbiology.net/), which contains three major components: data preprocessing, sequence mapping and results summarization. In the data preprocessing step, the adaptor sequences were trimmed and the low‐quality sequences such as low nucleotide complexity reads—homopolymer sequences or di‐ and tri‐nucleotide repeat sequences were removed. The processed sequences were then mapped against various databases including known human miRNA, human transcripts, followed by human genomic sequence. We also applied three different levels of error tolerance: 0 mismatch, 1 mismatch and 2 mismatches when aligning read sequence to databases. To be considered as detectable RNA species, the RNA has to have more than 10 mapped reads in at least 70% of the samples.

### 2.7 Validation of small RNASeq Results

The changes of miRNA concentrations determined by NGS were validated using the Taqman miRNA Assay kit. In brief, 2 μl of isolated RNA from individual samples was reverse transcribed using the TaqMan microRNA RT kit (Thermo Fisher, Waltham, MA). Real‐time qPCR amplification was performed on the BioRad C1000 Touch thermocycler. Enzyme was activated at 95°C for 10 minutes followed by 40 2‐step cycles of amplification at 95°C for 15 seconds and 60°C for 60 seconds. MiR‐16 (hsa‐miR‐16‐5p) was used as a normalization control for each assay, as miR‐16‐5p did not show significant concentration changes across samples.

### 2.8 Functional enrichment and network analyses

The biological impacts of PTL‐associated circulating miRNAs were assessed using predicted and validated miRNA‐mRNA interactions from miRTar database.^31^, ^32^ To focus on the possible functional changes in placenta, we filtered the miRNA target gene list with protein/transcript enriched in placenta based on human protein atlas.^33^ To gain a more complete view of perturbed network in PTL, we expanded the initial miRNA‐mRNA interaction with information from protein‐protein interaction databases.^34^, ^35^, ^36^ Functional enrichment analyses were conducted with DAVID (Database for Annotation, Visualization and Integrated Discovery) webtool.^37^ Cytoscape and KEGG pathway information were used to generate the network.^38^, ^39^ The process is illustrated in Figure S1.

## 3 RESULTS

### 3.1 Characteristics of study participants

Demographics of the 67 pregnant women (47 controls and 20 PTLs) who participated in the study are presented in Table S1. Samples from 11 preterm pregnancies and 11 matched normal pregnancies were selected for further processing of EVs and EV‐depleted plasma. Among the selected samples for EV analysis, all women reported previous pregnancies while half of the women had previously delivered a child and none of them had a previous preterm birth. The mean gestational age at delivery among the 11 PTL cases was 27.9 weeks and ranged between 23.3 and 33 weeks. The mean gestational age at delivery among the 11 controls was 39.9 weeks and ranged between 38.1 and 41.7 weeks. Characterization of SEC performance and EVs purified with the column is described in Supporting information and Figure S2.

### 3.2 General statistics for small RNA sequencing results

**Figure 1:**
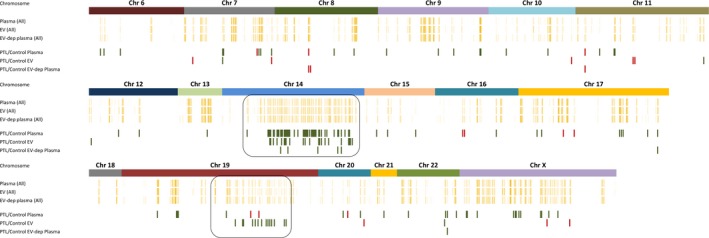
The overall distribution of miRNA independent of PTL (top panels) and PTL‐affected miRNAs (lower panels) in whole plasma, EV and EV‐depleted plasma (indicated on the left). The concentrations of all known miRNAs are displayed according to their chromosomal location (indicated on top). The strength of yellow colour of vertical bars represents the average concentrations observed in whole plasma, EV and EV‐depleted plasma (left) used in the study. The red (increased concentration in PTL compared to controls) and green (decreased concentration in PTL) colour bars represent the miRNA concentration changes associated with PTL. The two miRNA clusters in chromosome 14 and 19 are boxed. Even though the overall profiles of miRNA in different sample types are similar (upper panels), there are some miRNAs showing significant concentration differences associated with PTL. These concentration differences may be significant in one sample type (whole plasma) yet become insignificant in another (EV) demonstrating that the mechanisms by which the placenta releases them into circulation (either protein bound or packaged into exosomes) are differently affected by PTL

For sequencing results, we obtained on average about 12 million raw reads in whole plasma and 8.5 million in EV‐depleted plasma samples (Table 1). The average read count in the EV fractions is much higher because one control sample has more than 150 million reads. We did not observe any significant difference in raw read counts between controls and PTL samples. The number of observed and detectable miRNAs is highest for plasma and lowest for EV in both the control and PTL sets (Table 1). As expected, the overall miRNA profiles among the plasma, EV and EV‐depleted fractions are similar and 10 of the top 20 most abundant miRNAs are the same in all three sample types (Figure 1, Table S2). Among the detectable miRNAs (at least 10 mapped reads in 70% of samples), 481 of them are present in all three sample types (Figure S3). Interestingly, 15 unique miRNAs were detected in the EV‐depleted plasma and 9 unique miRNAs were identified in EV; these miRNAs may have been too dilute in the whole plasma and therefore below our detectable limit.

**Table 1:** General statistics for small RNA sequencing results

	Whole‐plasma control (47)	Whole‐plasma PTL (20)	EV control (11)	EV PTL (11)	EV‐dep plasma control (11)	EV‐dep plasma PTL(11)
Raw read count	12 900 538	11 024 622	23 129 552	9 374 019	8 509 117	8 516 263
Trimmed read count	9 479 818	8 306 794	18 135 858	7 105 360	6 581 375	6 582 738
Total miRNA mapped read	5 756 581	5 449 807	8 827 618	2 491 957	3 721 653	3 909 309
Number of observed miRNA (at least one mapped read)	1375	1313	998	966	1188	1111
Detectable miRNA (10 or more mapped reads in at least 70% of samples)	651	648	512	499	584	487

**Figure 2:**
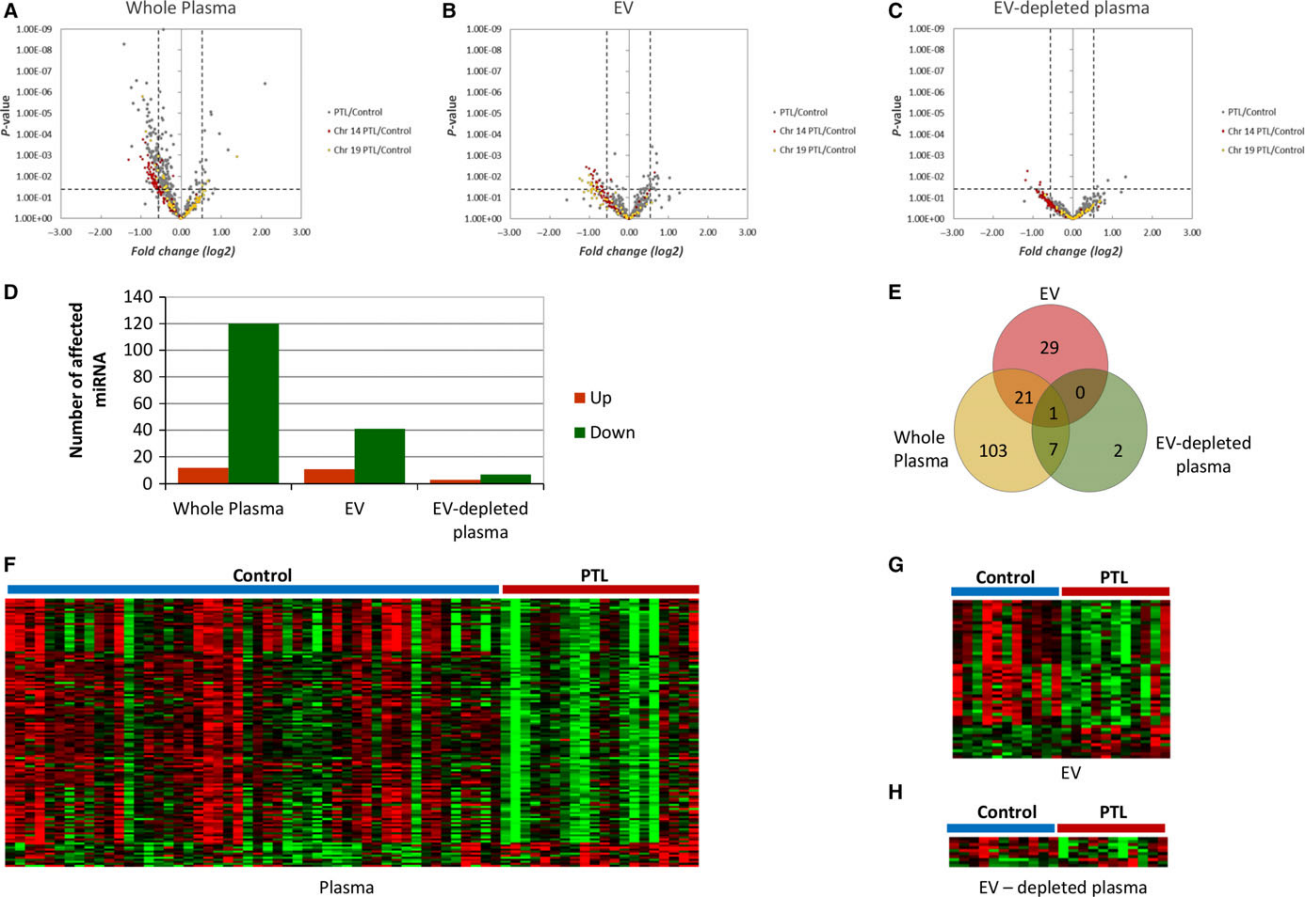
Circulating miRNA affected by PTL. The volcano plots show the effects of PTL on circulating miRNA in whole plasma (A), EV (B) and EV‐depleted plasma (C). The bar graph (D) and Venn diagram (E) show the number of affected miRNA in different sample types. Figures (F) (whole plasma), (G) (EV) and H (EV‐depleted plasma) are mean‐centred expression profiles of affected miRNAs. The patient conditions are indicated on top of the figures. The colours represent the miRNA concentrations that are either higher (red) or lower (green) than the average concentration of specific miRNA across all the samples

**Figure 3:**
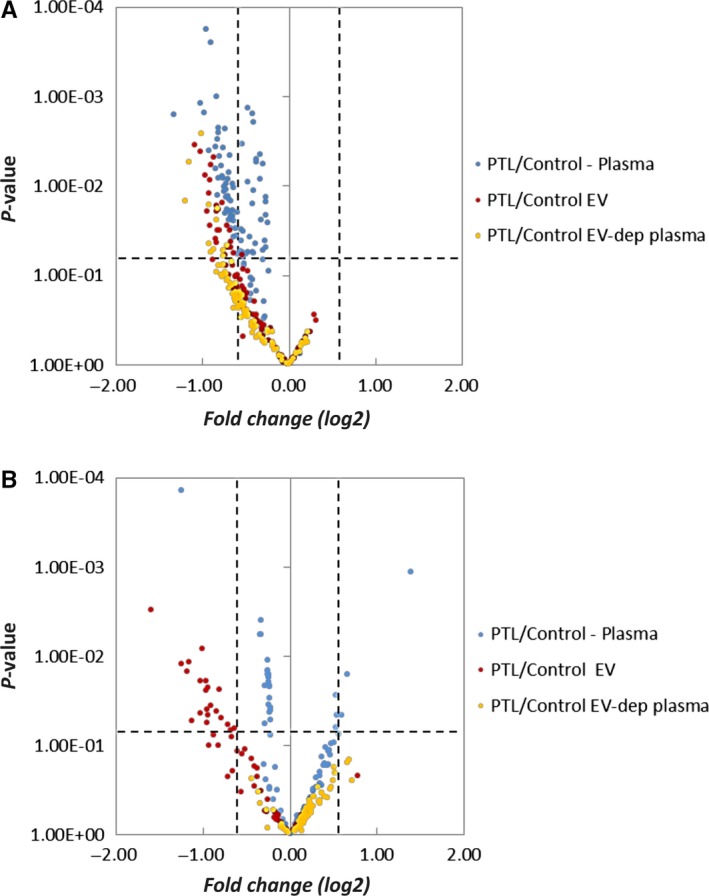
Volcano plots of miRNAs located in chromosome 14 (C14MC) (A) and chromosome 19 (C19MC) (B). The miRNA in whole plasma is indicated in blue dots, EV in red dots and EV‐depleted plasma in yellow dots

Even though the overall profiles of miRNA in different samples are similar, there are some miRNAs showing concentration differences associated with PTL (Figures 1, 2 and 3). Affected miRNAs in whole plasma, EV and EV‐depleted plasma are listed in Table 2. It is interesting that a large portion of the PTL‐affected RNAs in whole plasma and EV are from two major miRNA clusters, one on chromosome 14 and the other on chromosome 19 (Figures 1, 2A, B and C). The miRNAs in these clusters have been implicated in placenta development.^20^

**Table 2:** List of DEmiRNAs

miRNA ID	C14MC	C19CM	Whole plasma		EV		EV‐depleted plasma	
			Fold change (log2) PTL/control	*P*‐value	Fold change (log2) PTL/control	*P*‐value	Fold change (log2) PTL/control	*P*‐value
hsa‐miR‐100‐5p					−0.82	4.63E−02		
hsa‐miR‐1‐1‐3p			−0.86	1.76E−02				
hsa‐miR‐1179‐5p			−0.83	3.94E−06				
hsa‐miR‐1185‐1‐3p	X		−0.82	3.02E−03				
hsa‐miR‐1185‐1‐5p	X		−0.95	1.76E−04				
hsa‐miR‐1185‐2‐3p	X		−0.72	2.29E−03				
hsa‐miR‐1246‐5p			2.10	3.90E−07				
hsa‐miR‐1249‐3p			−1.23	3.81E−04			−1.04	3.46E−02
hsa‐miR‐1256‐3p			−0.61	4.34E−03				
hsa‐miR‐126‐3p			−0.63	1.09E−03				
hsa‐miR‐126‐5p			−0.62	5.66E−04				
hsa‐miR‐127‐3p	X		−0.97	1.53E−03				
hsa‐miR‐127‐5p	X		−0.75	1.16E−02	−0.94	1.91E−02		
hsa‐miR‐1277‐3p			−1.42	5.46E−09				
hsa‐miR‐1277‐5p			−1.20	3.42E−06				
hsa‐miR‐130a‐3p			−0.69	2.10E−03				
hsa‐miR‐130a‐5p			−0.62	1.15E−03				
hsa‐miR‐1343‐3p			−0.68	4.79E−03				
hsa‐miR‐135a‐1‐5p			−1.06	7.26E−06				
hsa‐miR‐136‐3p	X		−0.68	2.40E−02	−0.83	1.66E−02		
hsa‐miR‐136‐5p	X		−0.84	6.39E−03			−1.14	5.38E−03
hsa‐miR‐141‐3p					−0.81	7.49E−03		
hsa‐miR‐142‐3p			−0.65	1.94E−03				
hsa‐miR‐146b‐5p			−0.69	8.22E−05				
hsa‐miR‐153‐1‐3p			−0.61	5.55E−04				
hsa‐miR‐154‐3p	X		−0.75	3.77E−03			−0.92	4.46E−02
hsa‐miR‐154‐5p	X		−0.69	7.13E−03				
hsa‐miR‐155‐5p			−0.69	6.57E−04				
hsa‐miR‐181b‐2‐5p			−0.61	4.16E−04				
hsa‐miR‐181c‐3p			−0.66	3.01E−03				
hsa‐miR‐181c‐5p			−0.96	1.63E−06				
hsa‐miR‐181d‐5p			−0.77	1.98E−04				
hsa‐miR‐183‐5p			0.63	1.69E−03				
hsa‐miR‐186‐3p			−0.84	3.06E−04				
hsa‐miR‐192‐5p					0.71	2.26E−02		
hsa‐miR‐193a‐5p			0.96	9.50E−05				
hsa‐miR‐193b‐3p			0.70	1.60E−02				
hsa‐miR‐194‐1‐5p					0.63	3.55E−02		
hsa‐miR‐199a‐1‐5p			−0.89	7.18E−05				
hsa‐miR‐19b‐2‐3p			−0.72	2.15E−05				
hsa‐miR‐20a‐3p			−0.85	6.27E−06				
hsa‐miR‐214‐3p					0.69	4.40E−02		
hsa‐miR‐215‐5p			−0.79	2.07E−02				
hsa‐miR‐219a‐1‐5p			−0.84	1.37E−05				
hsa‐miR‐221‐3p			−0.61	6.16E−04				
hsa‐miR‐26a‐1‐5p			−0.76	1.17E−04				
hsa‐miR‐26a‐2‐3p			−0.60	2.91E−04				
hsa‐miR‐26b‐5p			−0.80	9.30E−06				
hsa‐miR‐299‐3p	X		−0.64	2.17E−02				
hsa‐miR‐299‐5p	X		−0.81	2.25E−03			−0.82	1.79E−02
hsa‐miR‐29a‐5p			−0.62	4.49E−04				
hsa‐miR‐29b‐2‐3p			−1.11	2.93E−07				
hsa‐miR‐301a‐3p			−0.87	3.85E−07				
hsa‐miR‐301b‐3p			−0.85	2.64E−05				
hsa‐miR‐30d‐3p			−0.61	5.87E−03				
hsa‐miR‐32‐3p			−0.75	8.85E−05				
hsa‐miR‐324‐5p			−0.62	1.07E−03				
hsa‐miR‐328‐3p			−0.77	1.29E−03				
hsa‐miR‐331‐3p			−0.74	1.09E−03				
hsa‐miR‐335‐3p			−0.82	4.57E−04				
hsa‐miR‐337‐3p	X		−0.71	1.83E−02	−0.83	1.91E−02		
hsa‐miR‐337‐5p	X		−0.69	2.41E−02	−0.87	4.76E−03		
hsa‐miR‐338‐3p			−0.80	5.37E−04			−0.86	4.24E−02
hsa‐miR‐33a‐3p					−0.67	2.14E−02		
hsa‐miR‐33a‐5p			−0.67	1.52E−02				
hsa‐miR‐340‐5p			−0.75	1.07E−04				
hsa‐miR‐3611‐3p			−0.73	1.13E−03				
hsa‐miR‐3617‐5p			−0.85	1.32E−04				
hsa‐miR‐365a‐3p			0.60	6.57E−03				
hsa‐miR‐3667‐5p			−0.67	2.29E−02				
hsa‐miR‐369‐3p	X		−0.68	1.90E−02	−0.96	7.59E−03		
hsa‐miR‐369‐5p	X		−0.63	2.48E−02	−0.90	2.78E−02		
hsa‐miR‐370‐3p			−0.81	8.12E−03				
hsa‐miR‐374a‐5p			−0.93	2.39E−06				
hsa‐miR‐374b‐3p			−0.79	9.37E−05				
hsa‐miR‐374b‐5p			−0.79	4.16E−05				
hsa‐miR‐376a‐1‐3p	X		−0.70	8.44E−03				
hsa‐miR‐376a‐1‐5p	X		−0.90	2.51E−04				
hsa‐miR‐376b‐3p	X		−1.01	1.19E−03	−0.71	2.75E−02		
hsa‐miR‐376c‐3p	X		−0.66	2.17E−02	−0.80	1.85E−02		
hsa‐miR‐376c‐5p	X		−1.32	1.58E−03				
hsa‐miR‐377‐3p	X		−0.82	1.26E−02	−0.89	5.77E−03	−1.19	1.46E−02
hsa‐miR‐378c‐5p					0.70	8.26E−04		
hsa‐miR‐379‐3p	X		−0.80	4.61E−03	−0.83	4.30E−02		
hsa‐miR‐379‐5p	X		−0.73	2.04E−02	−0.82	3.12E−02		
hsa‐miR‐380‐3p	X		−0.66	1.45E−02	−1.08	3.48E−03		
hsa‐miR‐381‐3p	X		−0.72	9.41E−03				
hsa‐miR‐382‐3p	X				−0.80	3.11E−02		
hsa‐miR‐409‐5p	X		−0.81	2.53E−03				
hsa‐miR‐410‐3p	X				−0.77	1.54E−02		
hsa‐miR‐411‐3p	X		−0.72	1.34E−02				
hsa‐miR‐411‐5p	X		−0.64	1.86E−02	−0.84	3.90E−02		
hsa‐miR‐421‐5p			−0.61	3.33E−03				
hsa‐miR‐431‐3p	X		−0.63	1.04E−02				
hsa‐miR‐431‐5p	X		−0.67	3.01E−02	−0.70	4.89E−02		
hsa‐miR‐432‐3p	X		−0.68	4.51E−03				
hsa‐miR‐4326‐5p					0.66	1.01E−02		
hsa‐miR‐450a‐1‐5p			−0.61	4.93E−03				
hsa‐miR‐4532‐5p			0.84	4.07E−02				
hsa‐miR‐454‐3p			−0.64	9.36E−05				
hsa‐miR‐4732‐5p			0.76	1.15E−05				
hsa‐miR‐483‐5p			1.18	5.55E−04			1.33	1.08E−02
hsa‐miR‐485‐3p	X		−0.66	1.90E−02				
hsa‐miR‐486‐1‐5p			0.75	9.07E−06			0.63	3.05E−02
hsa‐miR‐487a‐3p	X		−0.77	6.01E−03				
hsa‐miR‐487b‐3p	X		−0.68	1.38E−02	−0.67	4.82E−02		
hsa‐miR‐487b‐5p	X						−0.83	2.40E−02
hsa‐miR‐491‐5p			−0.74	8.20E−04				
hsa‐miR‐493‐3p	X		−0.74	1.01E−02				
hsa‐miR‐493‐5p	X		−0.72	1.08E−02				
hsa‐miR‐494‐3p	X		−0.76	7.83E−03				
hsa‐miR‐495‐3p	X		−0.75	8.20E−03	−0.67	4.24E−02		
hsa‐miR‐495‐5p	X		−0.64	9.50E−03				
hsa‐miR‐505‐5p					0.61	1.36E−02		
hsa‐miR‐512‐1‐5p		X			−0.85	4.14E−02		
hsa‐miR‐515‐1‐3p		X			−0.82	2.35E−02		
hsa‐miR‐515‐1‐5p		X			−0.97	2.45E−02		
hsa‐miR‐516b‐1‐5p		X			−0.91	3.62E−02		
hsa‐miR‐517a‐3p		X			−0.96	1.90E−02		
hsa‐miR‐517c‐3p		X			−1.04	1.92E−02		
hsa‐miR‐518b‐3p		X			−1.19	1.50E−02		
hsa‐miR‐518c‐3p		X			−1.03	4.42E−02		
hsa‐miR‐518f‐3p		X			−0.94	2.25E−02		
hsa‐miR‐518f‐5p		X	1.39	1.15E−03				
hsa‐miR‐5193‐3p			−0.64	2.46E−03				
hsa‐miR‐519d‐3p		X			−0.95	3.93E−02		
hsa‐miR‐520a‐3p		X	0.66	1.61E−02				
hsa‐miR‐520d‐5p		X			−1.01	8.36E−03		
hsa‐miR‐524‐5p		X			−0.94	4.56E−02		
hsa‐miR‐525‐5p		X			−1.26	1.22E−02		
hsa‐miR‐526b‐5p		X			−0.79	4.88E−02		
hsa‐miR‐539‐3p	X		−0.84	3.66E−03	−0.92	1.22E−02		
hsa‐miR‐545‐5p			−0.79	2.36E−05				
hsa‐miR‐548a‐1‐3p			−0.76	8.36E−03				
hsa‐miR‐548a‐3‐5p			−0.71	2.35E−03				
hsa‐miR‐548ax‐3p			−0.68	1.47E−03				
hsa‐miR‐548ax‐5p			−0.79	7.02E−04				
hsa‐miR‐551b‐3p			−0.71	2.24E−03	−0.74	1.45E−02		
hsa‐miR‐556‐3p			−0.73	6.56E−04				
hsa‐miR‐556‐5p			−0.85	4.70E−04				
hsa‐miR‐582‐5p			0.61	2.23E−02				
hsa‐miR‐589‐3p					0.71	2.26E−03		
hsa‐miR‐590‐3p			−1.25	6.34E−07	−0.68	2.98E−02		
hsa‐miR‐590‐5p			−0.76	4.93E−05				
hsa‐miR‐625‐5p			−0.61	2.70E−03				
hsa‐miR‐627‐3p			−0.63	3.78E−03				
hsa‐miR‐628‐5p			−0.75	2.27E−06				
hsa‐miR‐6516‐5p			−0.67	1.47E−03				
hsa‐miR‐654‐3p	X		−0.63	3.71E−02				
hsa‐miR‐655‐3p	X		−0.69	1.30E−02	−0.91	8.58E−03		
hsa‐miR‐656‐3p	X				−1.01	4.12E−03		
hsa‐miR‐664a‐3p			−0.66	5.82E−05				
hsa‐miR‐671‐5p			−0.64	2.55E−03	0.74	1.23E−02		
hsa‐miR‐6741‐3p			−0.62	1.52E−03				
hsa‐miR‐744‐3p			−0.65	1.24E−03				
hsa‐miR‐758‐3p	X		−0.91	4.00E−03				
hsa‐mir‐7641‐2					0.65	6.42E−03		
hsa‐miR‐877‐3p			−0.62	1.14E−02				
hsa‐miR‐889‐3p	X		−0.60	2.98E−02	−0.68	3.14E−02		
hsa‐miR‐92a‐2‐3p					0.73	9.10E−03		
hsa‐miR‐99b‐3p			−0.60	1.25E−02				

Of the 668 detectable miRNAs in whole plasma (631 shared plus 20 in control and 17 in PTL), 132 showed a significant concentration difference (≥1.5 change and *P*‐value ≤.05) between the preterm and normal gestation groups. Likewise, 51 of the 535 detectable miRNAs in EVs (476 shared plus 36 in control and 23 in PTL) had significant concentration changes between the preterm and normal gestation groups. Only 10 of the 585 detectable miRNA in the EV‐depleted plasma samples (486 shared plus 98 in control and 1 in PTL) had significant concentration changes between the two groups.

The chromosome 14 microRNA cluster (C14MC) is located at the imprinted, maternally expressed DLK‐DIO3 region on the human chromosome 14q32 and is the largest known miRNA cluster with 52 miRNA precursors (miRBase, http://www.mirbase.org) having the potential to be processed into 104 mature miRNAs.^40^, ^41^, ^42^ 95 of the mature miRNAs were represented by at least one mapped read across all sample types (whole plasma, EV and EV‐depleted plasma) in our analysis. The concentrations of miRNAs from the C14MC cluster were generally decreased in the PTL group compared to normal gestation group (Figures 1, 2A and 3A). The only PTL‐affected miRNA shared among all 3 sample types is hsa‐miR‐377‐3p, a miRNA that is known to be enriched in the placenta.

The chromosome 19 (C19MC) cluster is primate‐specific and consists of 50 miRNA precursors (miRBase, http://www.mirbase.org) that can produce 100 mature miRNAs and 91 of them were observed in our dataset. Our data showed that the concentrations of miRNAs from the C19MC cluster, like those in the C14MC cluster, were generally decreased in the PTL group compared to the normal gestation group, especially in the EV fraction (Figures 1, 2B and 3B). A study by Morales‐Prieto et al^43^ showed that expression of miRNAs within C19MC increases significantly from the first to the third‐trimester trophoblast, whereas that of C14MC members decreases.

### 3.3 qPCR validation of affected miRNAs

**Figure 4:**
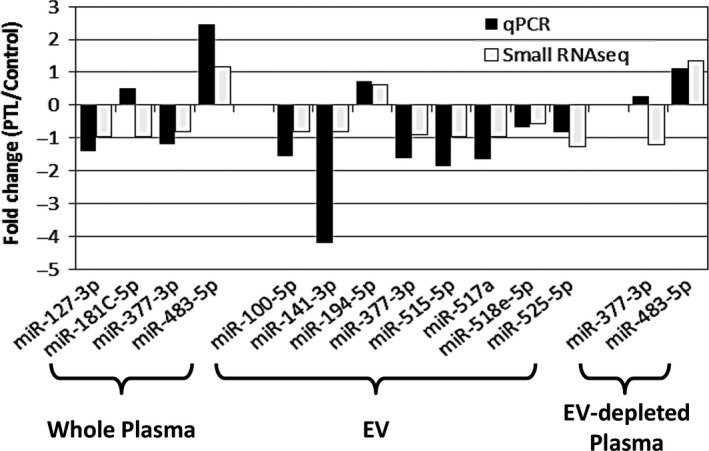
Validation of small RNA sequencing results. Some of the affected miRNAs determined by sequencing (open bars) were validated by qPCR (solid bars). The *X*‐axis indicates the identity of affected miRNA, and the *Y*‐axis is the fold changes in either cycle number (qPCR) or log2 transformed reads per million (RPM) adjusted read counts (small RNAseq)

Based on the RNASeq profiling results, we selected a handful of miRNAs to be verified by qPCR: hsa‐miR‐127‐3p and hsa‐miR‐181c‐5p for whole plasma; hsa‐miR‐100‐5p, hsa‐miR‐141‐3p, hsa‐miR‐194‐5p, hsa‐miR‐515‐5p, hsa‐miR‐517a‐3p, hsa‐miR518e‐5p and hsa‐miR‐525‐5p for EV‐associated; hsa‐miR‐377‐3p for all three sample types; and hsa‐miR‐483‐5p for whole plasma and EV‐depleted plasma. The qPCR and sequence results are in agreement, except hsa‐miR‐181c‐5p in whole plasma and hsa‐miR‐377‐3p in EV‐depleted plasma (Figure 4). These discrepancies between NGS and qPCR results may be due to low miRNA concentration or high sequence similarity with other family members. Nevertheless, our interplatform agreement is higher than previously reported expectations.^44^

## 4 DISCUSSION

For this study, we overcame several challenges associated with the profiling and analysis of extracellular miRNA and identified several circulating miRNAs (164 in total) whose concentrations were changed in PTL. The results feature many of the same PTL‐affected miRNAs in placenta or maternal plasma reported in prior studies; however, the direction of concentration change is not always in agreement.^45^, ^46^ Nevertheless, this is the first comprehensive characterization of RNA in whole plasma, EV and EV‐depleted plasma fractions in PTL and provides insight into the effect of PTL on the spectrum of circulating RNA and how they may be used as biomarkers for PTB. As we do not have earlier samples from the individuals we analysed, the study was limited to the characterization of circulating RNA profiles from women already symptomatic for PTL. It is possible that the labour itself affects the spectrum of circulating RNA; therefore, we need to be careful when interpreting the results.

One of the biggest challenges for circulating miRNA analysis is the inconsistency and irreproducibility of miRNA measurement results which we also see in the PTL literature. There are a number of reasons for this problem including sample preparation method and storage condition difference, low RNA concentration in the sample and measurement platform difference.^47^ For this study, we adapted several newly developed or improved methods with expectations to gain a more accurate and comprehensive profile of extracellular RNA. These methods include a new small RNA library construction protocol, a size exclusion chromatography (SEC)‐based EV purification method and a revised small RNA data analysis pipeline.^48^ One of the advantages for SEC‐based EV purification is the ability to analyse RNA in both EV and EV‐depleted fractions which allows the determination of RNA partition between the two compartments. With these improvements, we observed, on average, more than 1,300 different miRNAs in the whole‐plasma samples, 1,100 miRNAs in the EV‐depleted plasma and about 1,000 different miRNAs in the EV fraction. Our study also demonstrates results from this improved miRNA profiling approach provide better agreement with results from qPCR: 12 of the 14 miRNAs measured with qPCR aligned with our NGS results (Figure 4). The two that did not might be due to low miRNA concentration or high sequence similarity with other family members. The agreement between two different platforms further strengthens the small RNAseq findings and downstream analyses, such as network analysis.

### 4.1 Some miRNAs are enriched in EVs

Profiling both EV and EV‐depleted plasma allowed us to unequivocally determine the partition of specific miRNA between in and outside of EV in body fluid samples. Based on our sequencing results, the miRNA distribution between in and outside of EVs is different and more miRNAs are located outside of EVs (in EV‐depleted plasma; Table S3). There are at least four different reported methods for cells to sort miRNAs into EVs, especially for exosomes. These include protein‐mediated processes—one by neural sphingomyelinase 2 (nSMase2) 49 and the other by AGO2 50 and sequence‐dependent processes—uridine at 3′ end 51 or a sequence motif (GGAG) recognized by sumoylated heterogeneous nuclear ribonucleoproteins (hnRNPs).^52^ It is unclear whether the two protein‐mediated processes, nSMase 2 and AGO2 mediated miRNA sorting, are based on specific sequence motif(s). Examining the miRNA sequences between EV and EV‐depleted fractions, we could not find any common sequence motif(s) including the known exosomal miRNA‐associated motif GGAG.^52^ It is difficult for us to examine the non‐template addition of nucleotide(s) at the 3′ end due to the 4N adapter used in library preparation; however, the miRNAs showing higher concentrations in EVs have a higher per cent of U at the 3′ end compared to the EV‐depleted fraction (52% vs. 37%) (Figure S4A). In addition, the overall nucleotide composition of miRNAs preferentially packaged in EVs has a higher percentage of U while sequences outside of EVs have a higher percentage of G (Figure S4B). These findings suggest that some miRNAs showed higher concentrations in EVs, and these miRNAs are preferentially packaged into EVs by processes yet to be fully determined.

### 4.2 Circulating RNA may reflect the condition of placenta

Placenta‐associated mRNAs such as CSH1 (placental lactogen), CGB1 (chorionic gonadotropin beta 1) and PLAC1 (placenta‐specific protein 1), have been detected in maternal plasma, and their concentration changes in plasma can be used to reflect the health of the placenta.^53^, ^54^, ^55^ In this study, we did not detect sufficient reads mapped to various placenta‐enriched transcripts in either whole plasma, EV or EV‐depleted plasma. This is probably due to our library size selection step which focuses on RNA molecules that are around 20 nucleotides in length. A library with a larger insert size may reveal more reads that map to protein‐coding transcripts.

Nearly 50% of all PTL‐affected miRNAs, especially in the EV fraction, identified in the study belong to two large imprinted miRNA clusters, C19MC and C14MC that are known to be expressed by the placenta suggesting that circulating miRNA profile is a relevant resource to reflect the condition of placenta. The miRNAs from the C19MC region are expressed through the paternal allele and almost exclusively by the placenta. They have been shown to play an important role in placenta development through regulating cell proliferation, invasion and differentiation.^20^ The transcripts in the C14MC region are expressed through the maternal allele,^40^ are usually activated at critical developmental stages and are involved in controlling cell differentiation and fate in embryonic growth or placenta tissues.^56^ Although most of the miRNAs in the C14MC cluster are not exclusively expressed in the placenta, an analysis of healthy human tissues showed that some members of C14MC, for example hsa‐miR‐381, hsa‐miR‐154 and hsa‐miR‐377, are predominantly expressed in the placenta 57 and the concentrations of all three miRNAs are significantly decreased in PTL samples in our study.

In fact, most of the affected miRNAs identified in this study showed decreased concentrations in PTL samples (whole plasma, EV or EV‐depleted plasma). Some of these miRNAs have been reported to be associated with pregnancy‐related conditions. For example the plasma hsa‐miR‐517a level has been associated with ectopic pregnancy and preeclampsia,^58^, ^59^ and the plasma levels of several of the hsa‐miR‐520s in preeclampsia and molar pregnancy.^60^, ^61^, ^62^ However, the concentration changes of those miRNAs are different from the current report. Like what we observed in liver toxicity,^63^ there is a reverse correlation on concentration changes between plasma and tissue. For example, the level of hsa‐miR‐483‐5p is lower in placentas from PTL compared to full‐term pregnancies whereas we show an increased concentration in plasma of patients with PTL.^45^, ^46^ The levels of hsa‐miR‐154‐5p, hsa‐miR‐135a‐5p, hsa‐miR‐142‐3p, hsa‐miR‐136‐5p, hsa‐miR‐517a, hsa‐miR‐518b and hsa‐miR‐526b were increased in the placenta tissues of patients with PTL 64 whereas our data show a significant reduction in these miRNAs in circulation in patients with PTL. The reverse correlation of miRNA concentration changes between tissue and circulating miRNA has been reported, for example in a drug‐induced liver injury, the miR‐122 level is decreased in liver tissue but showed a significant increase in plasma. The increase in miR‐122 concentration in plasma probably is caused by miRNA released during hepatocyte death. In the case of PTL, we are not sure what caused the concentration difference between placenta and plasma. The dysfunction of placenta during PTL probably affects the release of miRNA into the extracellular environment. Further study is needed to resolve this discrepancy.

### 4.3 Key processes in placenta may be influenced by dysregulated miRNAs in PTL

**Figure 5:**
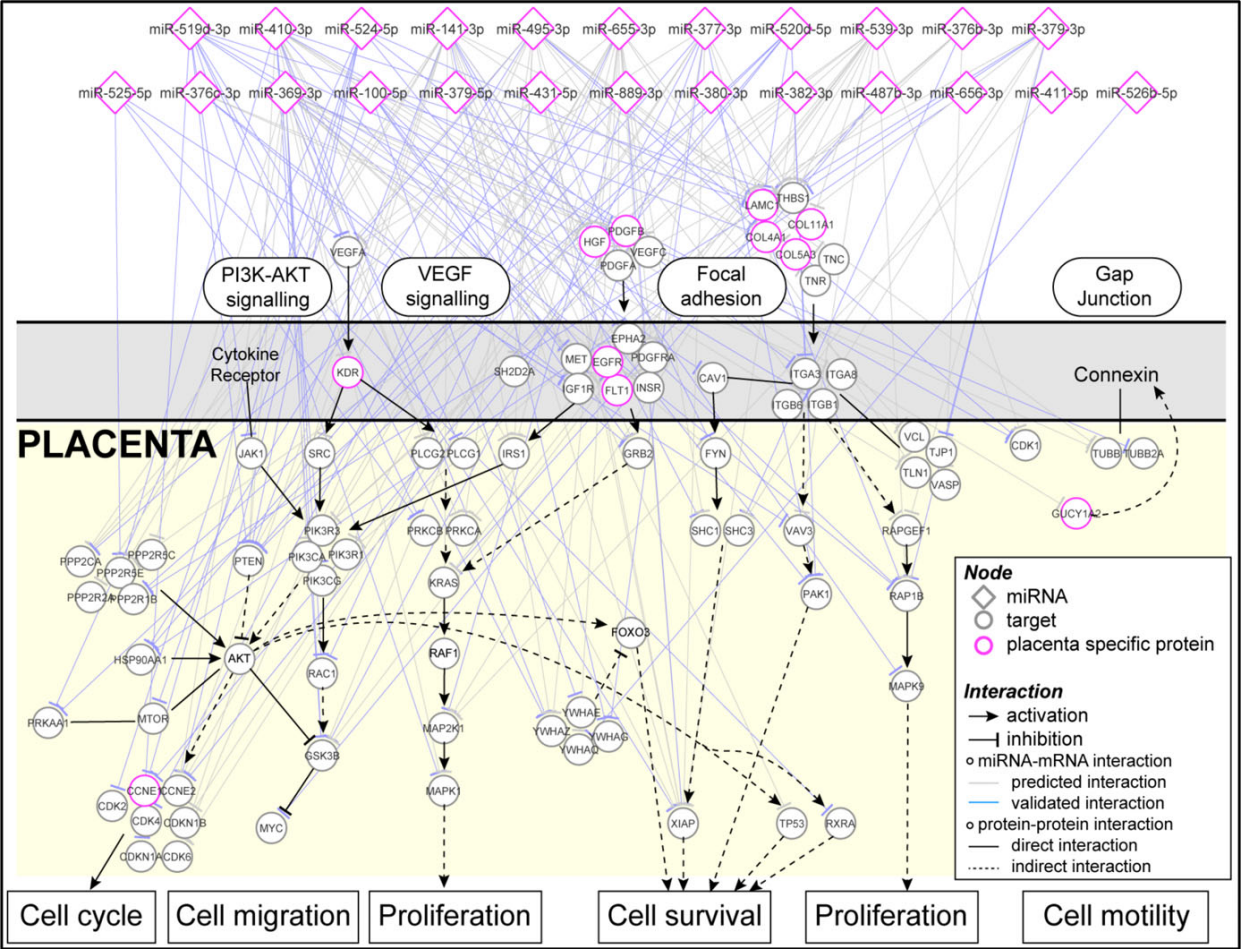
Schematic diagram of perturbed gene network in placenta that may be reflected by the changes of circulating miRNAs in PTL. The network is built based on the KEGG pathway maps: focal adhesion (hsa04510), PI3K‐Akt signalling pathway (hsa04151), gap junction (hsa04540) and VEGF signalling pathway (hsa04370) which were from results of the enrichment analysis of miRNA targets. The genes are indicated by circles and miRNAs by diamonds. The predicted miRNA‐mRNA interactions are indicated by light grey lines, and the blue lines are validated miRNA‐mRNA interactions. The identity of genes and miRNAs involved in the process are indicated, and the red colour indicates placenta‐enriched mRNAs and miRNAs

From the PTL‐affected miRNAs identified from whole plasma, EVs and EV‐depleted plasma, we conducted analysis to determine the perturbed networks represented by these dysregulated miRNAs in PTL. Based on TargetScan and miRTar databases,^31^, ^32^ the 127 differentially expressed miRNAs in PTL patient samples may interact with more than 11 000 different genes. To better reflect the changes of biological processes in placenta, we focused on the 70 miRNAs and 354 mRNAs that are enriched in placenta.^33^ We further expanded the 354 placenta‐enriched proteins with its first neighbour using protein‐protein interaction information,^34^, ^35^, ^36^ which allowed us to gain a more complete picture of the affected network. This produced a total of 3858 miRNA‐mRNA interactions between 70 miRNAs and 1,341 mRNAs. The 1341 protein‐coding mRNAs are mainly associated with processes involving signalling transduction and cell‐extracellular matrix interactions which are important processes involved in placenta and foetal development (Table 3). To illustrate the complexity of miRNA‐mRNA interactions, Figure 5 shows a detailed miRNA‐mRNA interaction network based on 4 cell proliferation‐related pathways: PI3K AKT signalling, VEGF signalling, focal adhesion and gap junction pathways derived from EV‐affected miRNA. The network contains 401 interactions between 54 miRNAs and 108 mRNAs. A number of miRNAs in the C14MC and C19MC regions are targeting key regulators. For example, *TP53* is targeted by hsa‐miR‐379‐5p (from C14MC), *MYC* is targeted by hsa‐miR‐487b‐3p and hsa‐miR‐494‐3p (from C14MC), *MET* is targeted by hsa‐miR‐369‐3p and hsa‐miR‐410‐3p (from C14MC), and *PTEN* by hsa‐miR‐519d‐3p, hsa‐miR‐520d‐3p and hsa‐miR‐524‐5p (from C19MC; Figure 5).

**Table 3:** List of putative circulating miRNA interacted pathways in placenta

KEGG ID	Pathway description	Plasma	EV	Depleted
hsa04510	Focal adhesion	5.81E−15	6.35E−13	7.79E−04
hsa04012	ErbB signalling pathway	3.44E−12	1.24E−13	3.49E−03
hsa04520	Adherens junction	1.12E−11	1.37E−13	1.07E−03
hsa04110	Cell cycle	2.05E−11	2.53E−11	2.25E−02
hsa04917	Prolactin signalling pathway	7.10E−11	2.53E−09	2.22E−02
hsa04151	PI3K‐Akt signalling pathway	1.01E−10	2.57E−10	7.82E−04
hsa04144	Endocytosis	4.92E−10	2.41E−09	7.03E−05
hsa04540	Gap junction	3.70E−09	1.34E−05	
hsa04350	TGF‐beta signalling pathway	5.50E−09	1.56E−08	1.79E−05
hsa04666	Fc gamma R‐mediated phagocytosis	1.13E−07	1.56E−08	5.99E−04
hsa04071	Sphingolipid signalling pathway	1.49E−06	4.17E−06	1.56E−03
hsa04664	Fc epsilon RI signalling pathway	2.60E−06	2.07E−05	
hsa04370	VEGF signalling pathway	1.21E−04	2.13E−05	
hsa04010	MAPK signalling pathway	2.27E−04	3.80E−04	6.45E−02
hsa04530	Tight junction	9.45E−04	3.28E−04	3.86E−03
hsa04512	ECM‐receptor interaction	2.84E−03		
hsa04068	FoxO signalling pathway	5.16E−08	4.43E−09	1.11E−02
hsa04152	AMPK signalling pathway	2.19E−03	5.64E−06	5.88E−02
hsa04914	Progesterone‐mediated oocyte maturation	6.89E−03	1.37E−03	

### 4.4 PTL may also affect the spectrum of other RNAs in circulation

Besides miRNA, our pipeline also reports other types of RNA in circulation. Like miRNA, we observed that the concentration of other RNAs including small nucleolar RNA (SnoRNA), piwi‐interacting RNA (piRNA) and long non‐coding RNA (lncRNA) was affected in patients with PTL (Table S4). The two affected SnoRNAs: SNORD22 and SNORD26 are encoded by small nucleolar RNA host gene 1 (SNHG1) on chromosome 11, and both of their concentrations increased in PTL plasma samples. Unlike miRNA, we know very little about the function of these RNAs; however, they probably also participate in foetal development. For example, the C14MC cluster is colocalized with a large cluster of SnoRNAs. One of them, the SNORD114 promotes cell cycle progression and overexpressing SNORD114 induces K562 and HCT116 cell proliferation. Even though we do not know their function, these PTL‐affected non‐coding RNAs identified in this study may lead to future functional studies on their involvement in normal foetal development.

In conclusion, we provide evidence for an altered profile of circulating RNA including miRNA and other small RNAs in the plasma from women with PTLs as compared to normal pregnancies and confirm the levels of some differentially expressed miRNAs (DEmiRNAs) in the whole plasma, EVs and EV‐depleted plasma by real‐time qPCR. We show that some of the EVs in plasma from pregnant women most likely originate from the placenta and make EV‐associated molecules a useful and relatively non‐invasive source of biomarkers for PTL. Further investigation with longitudinal and larger number of samples is required to validate a specific EV‐associated miRNA panel that can be used towards this goal.

## CONFLICT OF INTEREST

The authors declared that we have no conflict of interest.

## References

[1] Beck S , Wojdyla D , Say L , et al. The worldwide incidence of preterm birth: a systematic review of maternal mortality and morbidity. Bull World Health Organ. 2010;88:31‐38. 20428351 10.2471/BLT.08.062554 PMC2802437

[2] Morken N‐H . Preterm birth: new data on a global health priority. The Lancet. 2012;379:2128‐2130. 10.1016/S0140-6736(12)60857-5 22682451

[3] Stamey TA , Yang N , Hay AR , et al. Prostate‐specific antigen as a serum marker for adenocarcinoma of the prostate. N Engl J Med. 1987;317:909‐916. 2442609 10.1056/NEJM198710083171501

[4] Taketa K . α‐fetoprotein: reevaluation in hepatology. Hepatology. 1990;12:1420‐1432. 1701754 10.1002/hep.1840120625

[5] Verma P , Pandey RK , Prajapati P , Prajapati VK . Circulating MicroRNAs: potential and Emerging Biomarkers for Diagnosis of Human Infectious Diseases. Front Microbiol. 2016;7:1274. 27574520 10.3389/fmicb.2016.01274 PMC4983550

[6] Almeida MI , Reis RM , Calin GA . MicroRNA history: discovery, recent applications, and next frontiers. Mutat Res‐Fund Mol M. 2011;717:1‐8. 10.1016/j.mrfmmm.2011.03.009 21458467

[7] Arroyo JD , Chevillet JR , Kroh EM , et al. Argonaute2 complexes carry a population of circulating microRNAs independent of vesicles in human plasma. Proc Natl Acad Sci USA. 2011;108:5003‐5008. 21383194 10.1073/pnas.1019055108 PMC3064324

[8] Vickers KC , Palmisano BT , Shoucri BM , et al. MicroRNAs are Transported in Plasma and Delivered to Recipient Cells by High‐Density Lipoproteins. Nat Cell Biol. 2011;13:423‐433. 21423178 10.1038/ncb2210 PMC3074610

[9] Siriwardena AK , Mason JM , Mullamitha S , et al. Management of colorectal cancer presenting with synchronous liver metastases. Nat Rev Clin Oncol. 2014;11:446‐459. 24889770 10.1038/nrclinonc.2014.90

[10] Wang K , Zhang S , Marzolf B , et al. Circulating microRNAs, potential biomarkers for drug‐induced liver injury. Proc Natl Acad Sci USA. 2009;106:4402‐4407. 19246379 10.1073/pnas.0813371106 PMC2657429

[11] Akat KM , Moore‐McGriff DV , Morozov P , et al. Comparative RNA‐sequencing analysis of myocardial and circulating small RNAs in human heart failure and their utility as biomarkers. Proc Natl Acad Sci USA. 2014;111:11151‐11156. 25012294 10.1073/pnas.1401724111 PMC4121804

[12] Devaux Y , Vausort M , Goretti E , et al. Use of circulating MicroRNAs to diagnose acute myocardial infarction. Clin Chem. 2012;58:559. 22252325 10.1373/clinchem.2011.173823

[13] Tetta C , Ghigo E , Silengo L , et al. Extracellular vesicles as an emerging mechanism of cell‐to‐cell communication. Endocrine. 2013;44:11‐19. 23203002 10.1007/s12020-012-9839-0 PMC3726927

[14] Pereira L , Reddy AP , Alexander AL , et al. Insights into the multifactorial nature of preterm birth: proteomic profiling of the maternal serum glycoproteome and maternal serum peptidome among women in preterm labor. Am J Obstet Gynecol. 2010;202:555. 20413102 10.1016/j.ajog.2010.02.048

[15] Georgiou HM , Di Quinzio MKW , Permezel M , Brennecke SP . Predicting preterm labour: current status and future prospects. Dis Markers. 2015;2015:435014. 26160993 10.1155/2015/435014 PMC4486247

[16] Myntti T , Rahkonen L , Nupponen I , et al. Amniotic fluid infection in preterm pregnancies with intact membranes. Dis Markers. 2017;2017:8167276. 28167848 10.1155/2017/8167276 PMC5266802

[17] Weichert A , von Schöning D , Fischer T , Thomas A . Cervical sonoelastography and cervical length measurement but not cervicovaginal interleukin‐6 are predictors for preterm birth. Ultrasound Int Open. 2016;2:E83‐E89. 10.1055/s-0042-110317 PMC5032848 27689180

[18] Dulay AT , Buhimschi IA , Zhao G , et al. Compartmentalization of acute phase reactants interleukin‐6, C‐reactive protein and procalcitonin as biomarkers of intra‐amniotic infection and chorioamnionitis. Cytokine. 2015;76:236‐243. 25957466 10.1016/j.cyto.2015.04.014 PMC4824401

[19] Shahshahan Z , Rasouli O . The use of maternal C‐reactive protein in the predicting of preterm labor and tocolytic therapy in preterm labor women. Adv Biomed Res. 2014;3:154. 25221757 10.4103/2277-9175.137864 PMC4162078

[20] Mouillet J‐F , Ouyang Y , Coyne C , Sadovsky Y . MicroRNAs in placental health and disease. Am J Obstet Gynecol. 2015;213:S163‐S172. 26428496 10.1016/j.ajog.2015.05.057 PMC4592520

[21] Hromadnikova I , Kotlabova K , Ondrackova M , et al. Expression profile of C19MC microRNAs in placental tissue in pregnancy‐related complications. DNA Cell Biol. 2015;34:437‐457. 25825993 10.1089/dna.2014.2687 PMC4486149

[22] Hromadnikova I , Kotlabova K , Ondrackova M , et al. Circulating C19MC MicroRNAs in preeclampsia, gestational hypertension, and fetal growth restriction. Mediators Inflamm. 2013;2013:186041. 24347821 10.1155/2013/186041 PMC3848305

[23] Elovitz MA , Anton L , Bastek J , Brown AG . Can microRNA profiling in maternal blood identify women at risk for preterm birth? Am J Obstet Gynecol. 2015;212(782):e1‐e5. 10.1016/j.ajog.2015.01.023 25617732

[24] Gray C , McCowan LM , Patel R , et al. Maternal plasma miRNAs as biomarkers during mid‐pregnancy to predict later spontaneous preterm birth: a pilot study. Sci Rep. 2017;7:815. 28400603 10.1038/s41598-017-00713-8 PMC5429750

[25] Tian G , Yin X , Luo H , et al. Sequencing bias: comparison of different protocols of MicroRNA library construction. BMC Biotechnol. 2010;10:64. 20815927 10.1186/1472-6750-10-64 PMC2946280

[26] Toedling J , Servant N , Ciaudo C , et al. Deep‐sequencing protocols influence the results obtained in Small‐RNA sequencing. PLoS One. 2012;7:e32724. 22384282 10.1371/journal.pone.0032724 PMC3287988

[27] Mol EA , Goumans M‐J , Doevendans PA , et al. Higher functionality of extracellular vesicles isolated using size‐exclusion chromatography compared to ultracentrifugation. Nanomed Nanotechnol Biol Med. 2017;13:2061‐2065. 10.1016/j.nano.2017.03.011 28365418

[28] Blans K , Hansen MS , Sørensen LV , et al. Pellet‐free isolation of human and bovine milk extracellular vesicles by size‐exclusion chromatography. J Extracell Vesicles. 2017;6:1294340. 28386391 10.1080/20013078.2017.1294340 PMC5373680

[29] Théry C , Amigorena S , Raposo G , Clayton A . Isolation and characterization of exosomes from cell culture supernatants and biological fluids. Curr Protoc Cell Biol. 2006;Chapter 3: Unit 3.22. 10.1002/0471143030.cb0322s30 18228490

[30] Edgar R , Domrachev M , Lash A . Gene expression omnibus: NCBI gene expression and hybridization array data repository. Nucl Acids Res. 2002;30:207‐210. 11752295 10.1093/nar/30.1.207 PMC99122

[31] Chou CH , Chang NW , Shrestha S , et al. miRTarBase 2016: updates to the experimentally validated miRNA‐target interactions database. Nucleic Acids Res. 2016;44:D239‐D247. 26590260 10.1093/nar/gkv1258 PMC4702890

[32] Agarwal V , Bell GW , Nam JW , Bartel DP . Predicting effective microRNA target sites in mammalian mRNAs. Elife. 2015;4:e05005. 10.7554/eLife.05005 PMC4532895 26267216

[33] Uhlen M , Bjorling E , Agaton C , et al. A human protein atlas for normal and cancer tissues based on antibody proteomics. Mol Cell Proteomics. 2005;4:1920‐1932. 16127175 10.1074/mcp.M500279-MCP200

[34] Chatr‐Aryamontri A , Oughtred R , Boucher L , et al. The BioGRID interaction database: 2017 update. Nucleic Acids Res. 2017;45:D369‐D379. 27980099 10.1093/nar/gkw1102 PMC5210573

[35] Keshava Prasad TS , Goel R , Kandasamy K , et al. Human Protein Reference Database–2009 update. Nucleic Acids Res. 2009;37:D767‐D772. 18988627 10.1093/nar/gkn892 PMC2686490

[36] Bader GD , Betel D , Hogue CW . BIND: the biomolecular interaction network database. Nucleic Acids Res. 2003;31:248‐250. 12519993 10.1093/nar/gkg056 PMC165503

[37] Dennis G Jr , Sherman BT , Hosack DA , et al. DAVID: database for annotation, visualization, and integrated discovery. Genome Biol. 2003;4:P3. 12734009 PMC193660

[38] Kanehisa M , Furumichi M , Tanabe M , et al. KEGG: new perspectives on genomes, pathways, diseases and drugs. Nucleic Acids Res. 2017;45:D353‐D361. 27899662 10.1093/nar/gkw1092 PMC5210567

[39] Shannon P , Markiel A , Ozier O , et al. Cytoscape: a software environment for integrated models of biomolecular interaction networks. Genome Res. 2003;13:2498‐2504. 14597658 10.1101/gr.1239303 PMC403769

[40] Seitz H , Royo H , Bortolin M‐L , et al. A large imprinted microRNA gene cluster at the mouse Dlk1‐Gtl2 domain. Genome Res. 2004;14:1741‐1748. 15310658 10.1101/gr.2743304 PMC515320

[41] Gardiner E , Beveridge NJ , Wu JQ , et al. Imprinted DLK1‐DIO3 region of 14q32 defines a schizophrenia‐associated miRNA signature in peripheral blood mononuclear cells. Mol Psychiatry. 2012;17:827‐840. 21727898 10.1038/mp.2011.78 PMC3404364

[42] Ahmed MS , Aleksunes LM , Boeuf P , et al. IFPA Meeting 2012 Workshop Report II: epigenetics and imprinting in the placenta, growth factors and villous trophoblast differentiation, role of the placenta in regulating fetal exposure to xenobiotics during pregnancy, infection and the placenta. Placenta. 2013;34:S6‐S10. 23253784 10.1016/j.placenta.2012.11.020 PMC4682197

[43] Morales‐Prieto DM , Ospina‐Prieto S , Chaiwangyen W , et al. Pregnancy‐associated miRNA‐clusters. J Reprod Immunol. 2013;97:51‐61. 23432872 10.1016/j.jri.2012.11.001

[44] Mestdagh P , Hartmann N , Baeriswyl L , et al. Evaluation of quantitative miRNA expression platforms in the microRNA quality control (miRQC) study. Nat Meth. 2014;11:809‐815. 10.1038/nmeth.3014 24973947

[45] Mayor‐Lynn K , Toloubeydokhti T , Cruz AC , Chegini N . Expression profile of microRNAs and mRNAs in human placentas from pregnancies complicated by preeclampsia and preterm labor. Reprod Sci. 2011;18:46‐56. 21079238 10.1177/1933719110374115 PMC3343068

[46] Zhu XM , Han T , Sargent IL , et al. Differential expression profile of microRNAs in human placentas from preeclamptic pregnancies vs normal pregnancies. Am J Obstet Gynecol. 2009;200:e1‐e7. 10.1016/j.ajog.2008.12.045 19285651

[47] Lee I , Baxter D , Lee MY , et al. The importance of standardization on analyzing circulating RNA. Mol Diagn Ther. 2017;21:259‐268. 28039578 10.1007/s40291-016-0251-y PMC5426982

[48] Wu X , Kim T‐K , Baxter D , et al. sRNAnalyzer—a flexible and customizable small RNA sequencing data analysis pipeline. Nucleic Acids Res. 2017;45:12140‐12151. 29069500 10.1093/nar/gkx999 PMC5716150

[49] Kosaka N , Iguchi H , Hagiwara K , et al. Neutral sphingomyelinase 2 (nSMase2)‐dependent exosomal transfer of angiogenic microRNAs regulate cancer cell metastasis. J Biol Chem. 2013;288:10849‐10859. 23439645 10.1074/jbc.M112.446831 PMC3624465

[50] Frank F , Sonenberg N , Nagar B . Structural basis for 5′‐nucleotide base‐specific recognition of guide RNA by human AGO2. Nature. 2010;465:818‐822. 20505670 10.1038/nature09039

[51] Koppers‐Lalic D , Hackenberg M , Bijnsdorp IV , et al. Nontemplated nucleotide additions distinguish the small RNA composition in cells from exosomes. Cell Rep. 2014;8:1649‐1658. 25242326 10.1016/j.celrep.2014.08.027

[52] Villarroya‐Beltri C , Gutierrez‐Vazquez C , Sanchez‐Cabo F , et al. Sumoylated hnRNPA2B1 controls the sorting of miRNAs into exosomes through binding to specific motifs. Nat Commun. 2013;4:2980. 24356509 10.1038/ncomms3980 PMC3905700

[53] Takacs P , Jaramillo S , Datar R , et al. Placental mRNA in maternal plasma as a predictor of ectopic pregnancy. Int J Gynaecol Obstet. 2012;117:131‐133. 22342059 10.1016/j.ijgo.2011.12.011 PMC3327775

[54] Purwosunu Y , Sekizawa A , Farina A , et al. Cell‐free mRNA concentrations of CRH, PLAC1, and selectin‐P are increased in the plasma of pregnant women with preeclampsia. Prenat Diagn. 2007;27:772‐777. 17554801 10.1002/pd.1780

[55] Sekizawa A , Purwosunu Y , Farina A , et al. Prediction of pre‐eclampsia by an analysis of placenta‐derived cellular mRNA in the blood of pregnant women at 15‐20 weeks of gestation. BJOG. 2010;117:557‐564. 20121832 10.1111/j.1471-0528.2010.02491.x

[56] Lewis A , Mitsuya K , Umlauf D , et al. Imprinting on distal chromosome 7 in the placenta involves repressive histone methylation independent of DNA methylation. Nat Genet. 2004;36:1291‐1295. 15516931 10.1038/ng1468

[57] Liang Y , Ridzon D , Wong L , Chen C . Characterization of microRNA expression profiles in normal human tissues. BMC Genom. 2007;8:166. 10.1186/1471-2164-8-166 PMC1904203 17565689

[58] Kotlabova K , Doucha J , Hromadnikova I . Placental‐specific microRNA in maternal circulation–identification of appropriate pregnancy‐associated microRNAs with diagnostic potential. J Reprod Immunol. 2011;89:185‐191. 21513988 10.1016/j.jri.2011.02.006

[59] Zhao Z , Zhao Q , Warrick J , et al. Circulating microRNA miR‐323‐3p as a biomarker of ectopic pregnancy. Clin Chem. 2012;58:896‐905. 22395025 10.1373/clinchem.2011.179283 PMC3694411

[60] Miura K , Hasegawa Y , Abe S , et al. Clinical applications of analysis of plasma circulating complete hydatidiform mole pregnancy‐associated miRNAs in gestational trophoblastic neoplasia: a preliminary investigation. Placenta. 2014;35:787‐789. 25016558 10.1016/j.placenta.2014.06.004

[61] Hasegawa Y , Miura K , Furuya K , et al. Identification of complete hydatidiform mole pregnancy‐associated microRNAs in plasma. Clin Chem. 2013;59:1410‐1412. 23818445 10.1373/clinchem.2013.206391

[62] Hromadnikova I , Kotlabova K , Doucha J , et al. Absolute and relative quantification of placenta‐specific micrornas in maternal circulation with placental insufficiency‐related complications. J Mol Diagn. 2012;14:160‐167. 22251611 10.1016/j.jmoldx.2011.11.003

[63] Wang K , Zhang S , Marzolf B , et al. Circulating microRNAs, potential biomarkers for drug‐induced liver injury. Proc Natl Acad Sci U S A. 2009;106:4402‐4407. 19246379 10.1073/pnas.0813371106 PMC2657429

[64] Montenegro D , Romero R , Kim SS , et al. Expression patterns of microRNAs in the chorioamniotic membranes: a role for microRNAs in human pregnancy and parturition. J Pathol. 2009;217:113‐121. 18991333 10.1002/path.2463 PMC4160233

