# Supplementary material for: Extracellular vesicle RNAs reflect placenta dysfunction and are a biomarker source for preterm labour
Source: J Cell Mol Med. 2018 Mar 8;22(5):2760–73. doi: 10.1111/jcmm.13570 (PMC5908130; doi:10.1111/jcmm.13570)
Supplement: Supplementary file 1 [file JCMM-22-2760-s001.docx]

**Supporting Methods**

**Preparation of EV and EV-depleted plasma.** Eluate (~500 µl for each fraction) from qEV column was collected in microfuge tubes individually. The protein contents and concentration for each fraction were assessed by protein gel electrophoresis and Bradford assay (BioRad, Hercules, CA). The EV fractions (usually from fraction 7 to 10) and EV-depleted fractions (fractions 12 to 32) were pooled and concentrated to ~100 ul using Amicon 10K centrifugation filters (EMD Millipore, Billerica, MA) spun in a swing-bucket rotor at 4000 x g at 4^o^C for 20 minutes. To test the loss of miRNA through Amicon 10K spin-column, 100ul whole plasma was put onto the spin-column and RNA was isolated from the flow through. The concentration of two highly abundant miRNAs in plasma, miR-16-5p and miR-21-5p, were measured from both the flow through and whole plasma (S5 Fig).

**Supporting Results**

**Characterization of EVs purified with SEC**

We characterized SEC performance for EV purification based on overall blood protein distribution in different eluate fractions (assessed by gel electrophoresis), and transmission electron microscopy imaging of purified EVs. The overall blood protein distribution pattern is similar to prior reports [[1](#_ENREF_1)]; most of the blood proteins are not in the EV fraction (S2A Fig). The western blotting results also indicate that some of the known miRNA associated protein complexes such as Ago2 and ApoA1 (a component of HDL) are not in the EV fractions (S2B Fig). The electron micrographs show vesicle size of about 100 nm with typical donut shape (S2C Fig). These findings suggest that the EV fractions we collected through SEC have very little protein contamination and contain particles including exosomes and microvesicles (S2 Fig).

**Other affected RNAs in circulation**

Besides miRNA, our pipeline also reports other types of RNA in circulation. Like miRNA, we observed that the concentration of other noncoding RNAs including small nucleolar RNA (SnoRNA), piwi-interacting RNA (piRNA) and long non-coding RNA (lncRNA) were affected in patients with PTL (S4 Table). The two affected SnoRNAs: SNORD22 and SNORD26 are encoded by small nucleolar RNA host gene 1 (SNHG1), and both of their concentrations increased in PTL**.**

**Supporting Figure 1.** The flowchart of functional analysis on PTB affected circulating miRNAs.


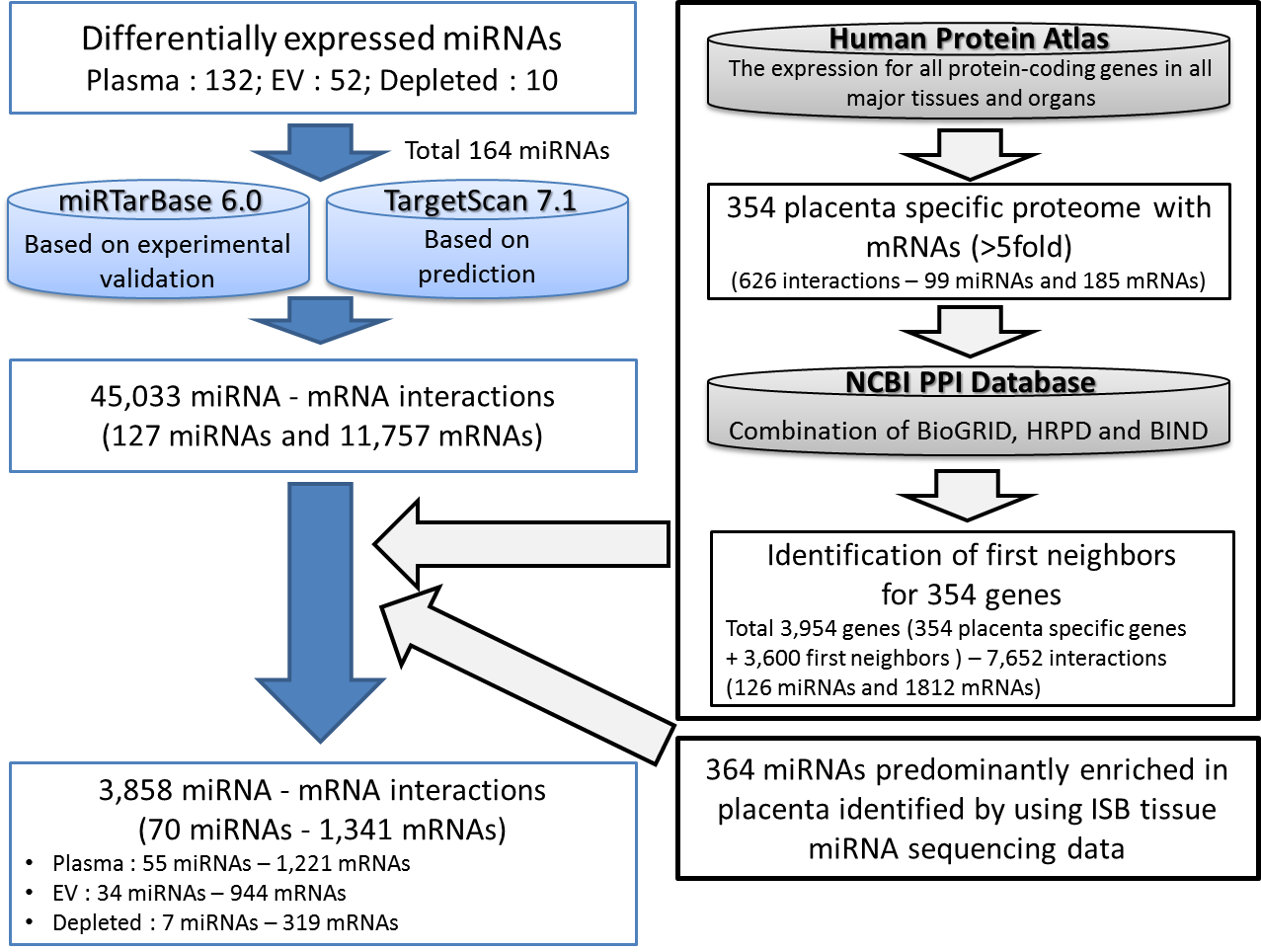


**Supporting Figure 2.** The quality of EV purification. Most of the proteins in plasma are not in the EV fraction based on protein concentration (A) and protein gel with coomassie blue staining (B). Western analysis also shows the known miRNA binding proteins such as Ago2 and ApoA1 (part of the HDL complex) are not in the EV fractions (B). The purified EVs showed typical exosome size and shape under transmission electron microscopy (C). Black bar in figure 2C indicates 100 nm.


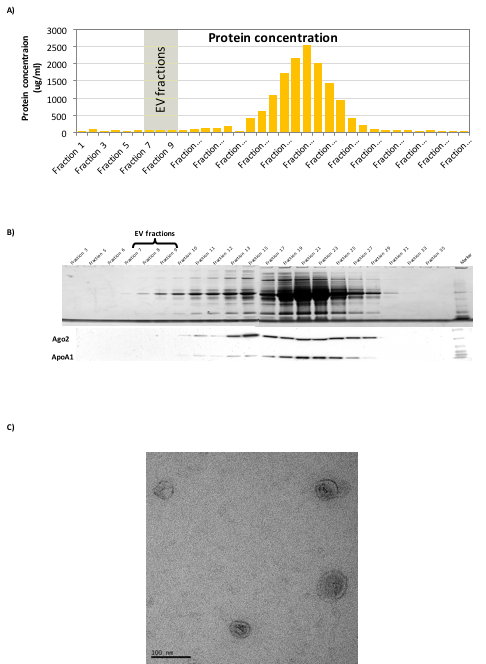


**Supporting Figure 3.** Comparing the number of detectable miRNAs in the three sample types, unassociated with disease. The whole plasma has the most number of detectable miRNAs, followed by EV-depleted plasma and EV. Most of the miRNAs are shared among the three sample types.

**Plasma**

**EV-depleted plasma**

**EV**

45

9

89

53

15

481

**Supporting Figure 4** Comparing the difference of miRNA sequences between in and outside of EV. A higher fraction of miRNAs preferentially packaged in EV has U at its 3’ end (57%) compared to sequences outside of EV (38%) (A). The overall nucleotide composition for miRNAs preferentially packaged in EV also has a higher content of U while sequences outside of EV have higher content of G.

A)


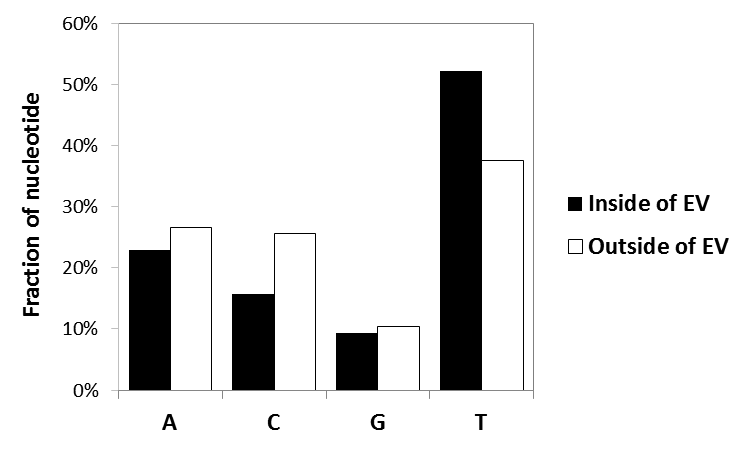


B)


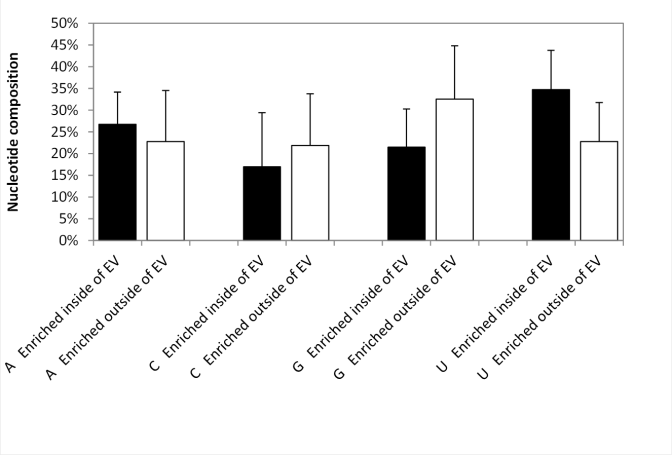


**Supporting Figure 5.** Using qPCR to assess the loss of miRNA through Amicon 10K spin column. Two highly abundant miRNAs, miR-16-5p and miR-21-5p were used to determine the level of miRNA that may have passed through the Amicon 10K spin column. Y axis represents the level of miRNA determined by qPCR (40-Ct) and the sample type is indicated in X-axis.

**Supporting Table 1.** Patient information. Abbreviations as follows: C-sec = cesarean section; VAG = vaginal birth; IOL = induction of labor; DC/DA = dichorionic diamniotic; MC/DA = monochorionic diamniotic; MC/MA = monochorionic monoamniotic; AROM = artificial rupture of membranes; SROM = spontaneous rupture of membranes; PROM = premature rupture of membranes; APH = antepartum haemorrhage; dem. 6 wks = fetal demise at 6 weeks gestation, second baby delivered at full term. Control samples CON1, CON21, and CON26 were removed from study analysis because gestational age at delivery did not meet criteria for full term pregnancy.

| Sample ID | BMI | Gest Age at Blood Collec(completed weeks + days) | Gest Age at Del (completed weeks + days) | Gravidity | Parity | Previous PTB (0/1) | Birth method | Induc-tion | Twins | Fetal Gender | PROM | APH | Meds | Steroids | Comments | Ethnicity | NGS | | |
| --- | --- | --- | --- | --- | --- | --- | --- | --- | --- | --- | --- | --- | --- | --- | --- | --- | --- | --- | --- |
|  |  |  |  |  |  |  |  |  |  |  |  |  |  |  |  |  | **Plasma** | **EV** | **EV dep plasma** |
| CON1 | 30 | 28+7 | 36+5 | 4 | 0 | 0 | C-sec | NO | 0 | M | AROM | 0 | Oxytocin, lactated ringers (LR), morphine, Narcon, |  | Removed from analysis due to gestational age at delivery. | Caucasian | X |  |  |
| CON2 | 37 | 28+2 | 39+3 | 6 | 2 | 1 | VAG | NO | dem.  6 wks | F | AROM | 0 | Oxytocin, DIclofenac | Synthroid | (Hypothyroid)-Eltroxin | Caucasian | X |  |  |
| CON3 | 22 | 28+5 | 39+2 | 2 | 1 | 0 | C-sec | NO | 0 | F | AROM | 0 |  |  | Hyperbilirubinemia | Asian | X |  |  |
| CON4 | 26 | 28+5 | 37+6 | 4 | 1 | 1 | VAG | IOL | 0 | F | AROM | 0 | PenG |  | Group B Strep positive (GBS+) |  | X |  |  |
| CON5 | 26 | 28+4 | 39+1 | 1 | 0 | 0 | C-sec | NO | 0 | F | 0 | 0 | synthroid | Synthroid | Hypothyroid | Caucasian | X |  |  |
| CON6 | 28 | 27+1 | 38+3 | 1 | 0 | 0 | VAG | NO | 0 | M | 0 | 0 |  | Synthroid | Type 2 diabetes | Asian | X |  |  |
| CON7 | 30 | 29+6 | 41 | 1 | 0 | 0 | C-sec | IOL | 0 | F | 0 | 0 | PenG |  | GBS+ | Asian | X |  |  |
| CON8 | 34 | 25+7 | 38+2 | 2 | 0 | 1 | C-sec | NO | 0 | M | 0 | 0 | Zoloft |  | GBS+ ,Gest diab. placenta previa, Endometriosis, | Caucasian | X |  |  |
| CON9 | 27 | 27+2 | 41 | 3 | 0 | 0 | C-sec | NO | 0 | M | 0 | 1 |  | Synthroid | GBS+ , cyst of thyroid, | Caucasian | X |  |  |
| CON10 | 31 | 27+6 | 38+1 | 2 | 1 | 0 | VAG | NO | 0 | F | 0 | 0 | PenG, Gravol, ceFAZolin, morphine, |  | oral Herpes, GD non-insulin | Caucasian | X | X | X |
| CON11 | 21 | 27+3 | 40+3 | 1 | 0 | 0 | VAG | IOL | 0 | F | AROM | 0 |  |  |  | Caucasian | X | X | X |
| CON12 | 30 | 28+6 | 37+6 | 3 | 1 | 1 | VAG | NO | 0 | M | 0 | 0 |  |  |  | Caucasian | X |  |  |
| CON13 | 31 | 28+7 | 40+2 | 2 | 0 | 0 |  | NO | 0 |  | 0 | 0 |  |  | Did not deliver at Mt. Sinai Hospital | Caucasian | X |  |  |
| CON14 | 27 | 27+4 | 39+3 | 3 | 1 | 1 | VAG | NO | 0 | M | 0 | 0 |  |  | GBS+, previous IUGR pregnancy | Asian | X |  |  |
| CON15 | 29 | 26+3 | 38+4 | 1 | 0 | 0 | VAG | NO | 0 | M | 0 | 0 |  |  |  | Caucasian | X |  |  |
| CON16 | 25 | 27+4 | 38+6 | 2 | 1 | 0 | VAG | NO | 0 | M | 0 | 0 |  |  |  | Caucasian | X |  |  |
| CON17 | 25 | 28 | 38+2 | 2 | 1 | 0 | C-sec | NO | 0 | F | 0 | 0 |  |  | 2009-fetal death-therapeutic abortion | other | X |  |  |
| CON18 | 30 | 28+3 | 39+0 | 2 | 1 | 0 | VAG | NO | 0 | F | SROM | 0 | home med.-synthroid, ventolin |  | GBS+, Gestational diabetes (GDM)-diet, Hypothyroid, asthma | Black | X | X | X |
| CON19 | 28 | 28+7 | 40+1 | 2 | 1 | 0 | VAG | NO | 0 | F | AROM | 0 |  |  | Pregnancy Induced Hypertension (PIH) | Caucasian | X |  |  |
| CON20 | 26 | 28+5 | 40+4 | 4 | 2 |  | VAG | NO | 0 | M | AROM | 0 |  |  |  | Caucasian | X | X | X |
| CON21 | 35 | 28+3 | 36 | 5 | 2 | 1 | VAG | IOL | 0 | F | 0 | 0 | PenG |  | PIH, GBS+, ovarian cyst (non cancer), pneumonia? Removed from analysis due to gestational age at delivery. | Asian | X |  |  |
| CON22 | 35 | 28+4 | 40+1 | 2 | 1 | 0 | VAG | NO | 0 | M | 0 | 0 | Tylenol, heparin, Diclofenac(anti inflam) |  |  | Caucasian | X | X | X |
| CON23 | 36 | 27+3 | 38 | 2 | 0 | 0 | C-sec | IOL | 0 | F | AROM | 0 |  | Synthroid | Hypothyroid , GDM-diet, PIH | Caucasian | X |  |  |
| CON24 | 29 | 28+5 | 37+1 | 1 | 0 | 0 | VAG | NO | 0 | M | 0 | 0 | Naloxon, gravol |  |  |  | X |  |  |
| CON25 | 26 | 28+3 | 40+4 | 2 | 1 | 0 | VAG | NO | 0 | F | 0 | 0 |  |  |  | other | X | X | X |
| CON26 | 33 | 28 | 36+2 | 1 | 0 | 0 | VAG | NO | 0 | F | 0 | 0 | Tylenol, heparin,hydrocortison, nitrogliceri | Synthroid | Hypothyroid , GDM-diet, PIH , IUGR  Removed from analysis due to gestational age at delivery. | Asian | X |  |  |
| CON27 | 23 | 27+4 | 38+6 | 3 | 1 | 0 | VAG | NO | 0 | F | AROM | 0 | Oxytocin |  |  | Caucasian | X | X | X |
| CON28 | 26 | 28+5 | 38+5 | 1 | 0 | 0 | C-sec | IOL | 0 | F | AROM | 0 | Oxytocin |  | nuchal cord 2x (cord around baby neck) | Caucasian | X |  |  |
| CON29 | 20 | 29 | 40+6 | 2 | 1 | 0 | VAG | IOL | 0 | F | AROM | 0 |  |  | small hemorrhoids | Caucasian | X |  |  |
| CON30 | 26 | 29+6 | 37+4 | 1 | 0 | 0 | VAG | NO | 0 | M | 0 | 0 |  |  |  | Caucasian | X |  |  |
| CON31 | 20 | 27+2 | 39+5 | 1 | 0 | 0 | VAG | NO | 0 | M |  | 0 | Oxytocine,Diclofenac,hydromorphon | hydrocortisone-zinc -topical | Endometriosis, hypothyroidism | Asian | X |  |  |
| CON32 | 27 | 26+7 | 40+4 | 1 | 0 | 0 | VAG | NO | 0 | M |  | 0 | Oxytocin |  |  | Asian | X |  |  |
| CON33 | 32 | 27+2 | 38+2 | 2 | 1 | 0 | C-sec | NO | 0 | M |  | 0 | Oxytocine,ceFazolin(antibiotic) |  |  | Caucasian | X |  |  |
| CON34 | 30 | 28+5 | 40+2 | 1 | 0 | 0 | VAG | NO | 0 | F | AROM | 0 | Oxytocin/epidural |  |  | Caucasian | X |  |  |
| CON35 | 29 | 28+6 | 38+1 | 5 | 2 | 0 | C-sec | NO | 0 | M |  | 0 |  |  | hypothyr, asthma, osteoarth, GEDR, ↓ amniotic fluid at 33+5 | Asian | X | X | X |
| CON36 | 26 | 29 | 41+1 | 1 | 0 | 0 | VAG | IOL | 0 | M | AROM | 0 | Oxytocin, prostin geal, acetaminophen, Diclofenac |  | Hypothyroid ,abnormal FHR, fetal deceleration | Asian | X | X | X |
| CON37 | 23.5 | 28 | 41+4 | 2 | 1 | 0 | C-sec | IOL | 0 | M | AROM | 0 | oxytocin/epidural/Antibiotic |  |  | Asian | X |  |  |
| CON38 | 29 | 28+3 | 40 | 1 | 0 | 0 | VAG | NO | 0 | M | AROM | 0 | Oxytocin |  | GDM diet cont. | Caucasian | X | X | X |
| CON39 | 24 | 28+2 | 39+2 | 3 | 2 | 0 | VAG | NO | 0 | F | SROM | 0 | Epidural |  |  | Caucasian | X |  |  |
| CON40 | 24 | 24+1 | 40+6 | 4 | 0 | 1 | C-sec | IOL | 0 | F | AROM | 0 | Oxytocin, LR, Pen G, |  | GBS+, 17 week therapeutic abortion -Trisomy21 | Caucasian | X |  |  |
| CON41 | 30 | 25+3 | 38+4 | 3 | 2 | 0 | C-sec | NO | 0 | M | AROM | 0 |  |  |  | Black | X |  |  |
| CON42 | 21 | 28+3 | 38+5 | 2 | 1 | 0 | C-sec | NO | 0 | M | AROM | 0 | Azathioprine |  | Crohn's syndrom :home med-Azathioprine | Caucasian | X |  |  |
| CON43 | 40 | 26+5 | 41 | 1 | 0 | 0 | VAG | IOL | 0 | F | AROM | 0 | epidural, tylenol, Diclofenac, colace |  | Asthma,PCO | Caucasian | X |  |  |
| CON44 | 29 | 28+1 | 41+5 | 1 | 0 | 0 | VAG | NO | 0 | F | AROM | 0 | gel, Oxytocin, LR, Tylenol, Colace, Diclofenac-after |  |  | Caucasian | X | X | X |
| CON45 | 24 | 26+1 | 40+5 | 1 | 0 | 0 | C-sec | NO | 0 | F | 0 | 0 | Oxytocin, LR, Tylenol, Colace,ZoFran, Naloxon |  | Sign of maternal infection | Caucasian | X |  |  |
| CON46 | 28 | 28 | 39+2 | 2 | 1 | 0 | VAG | NO | 0 | F | AROM | 1 | Epidural, Diclectin, |  | GBS(+), Asthma | Asian | X |  |  |
| CON47 | 30 | 27+5 | 39+6 | 2 | 0 | 0 | C-sec | NO | 0 | M | SROM | 0 | Oxytocin, Epidural, Gravol , Narcan, | Synthroid | Hypothyroid | Caucasian | X |  |  |
| CON48 | 30 | 27+4 | 39+2 | 2 | 0 | 0 | C-sec | IOL | 0 | M | 0 | 0 | Oxytocin/epidural | Synthroid | Hypothyroid | Caucasian | X |  |  |
| CON49 | 28 | 27+5 | 38+6 | 1 | 0 | 0 | C-sec | NO | 0 | F | AROM | 0 | Oxytocin, antibiotics |  | Meningitis due to Herpes virus | Caucasian | X |  |  |
| CON50 | 25 | 27+6 | 39+5 | 3 | 0 | 0 | C-sec | IOL | 0 | F | ROM 2 d. bef | 0 | Oxytocin,nitroglicerine, diclectin in June(days before delivery) |  |  | Caucasian | X |  |  |
|  |  |  |  |  |  |  |  |  |  |  |  |  |  |  |  |  |  |  |  |
| PTL1 | 25 | 30+1 | 30+1 | 2 | 0 | 0 | c-sec | NO | DC/DA | M/M | 0 | 0 | Celeston, MgSO4 |  |  | Asian | X |  |  |
| PTL2 | 24.6 | 27+5 | 27+6 | 1 | 0 | 0 | Vag | NO | 0 | F | 0 | 0 | MgSO4, PenG, Nifedipile, Celeston 2xDICLOFENAC, nalaxon, hydromorphone, anti-inflamatory drug | Hydrocortison-topical, Bethametasone | GBS +, placenta abruption |  | X | X | X |
| PTL3 | 38 | 33+2 | 33+2 | 3 | 1 | 0 | Vag | NO | 0 | F | 0 | 1 | MgSO4, PenG |  | Fetal anomaly |  | X |  |  |
| PTL4 | 27 | 30+4 | 30+4 | 1 | 0 | 0 | Vag | NO | 1 (A-live, B dead) | F | 1 | 0 | MgSO4, PenG, Gravol, Celest -2X, DICLOFENAC, anti-inflamatory drug |  | GBS+, 1-twin dead-fetal anomaly | Caucasian | X |  |  |
| PTL5 | 22 | 23+6 | 23+6 | 2 | 1 | 1 | Vag | NO | 0 | F | 0 | 0 | MgSO4, Gravol, Morphine, Celeston x2 |  |  |  | X |  |  |
| PTL6 | 25 | 33 | 33 | 2 | 2 | 0 | Vag | NO | 0 | M | AROM | 0 | Celeston, |  |  |  | X | X | X |
| PTL7 | 30 | 24+0 | 24+3 | 2 | 1 | 0 | Vag | NO | 0 | M | 0 | 0 | Celeston 2x , MgSO4, Morphin, |  |  | Asian(Indian) | X | X | X |
| PTL8 | 32 | 33 | 33+1 | 3 | 1 | 0 | Vag | NO | 0 |  | 0 | 0 | Celeston, PenG, Gravol, Morphin, |  |  | Caucasian | X |  |  |
| PTL9 | 22 | 28+1 | 28+1 | 1 | 0 | 0 | Vag | NO | DC/DA | F/F | 0 | 0 | Celoston, Nitro, Clindamycin, |  | HPV -3 months ago | Caucasian | X |  |  |
| PTL10 | 19 | 26+5 | 26+5 | 3 | 0 | 0 | Vag | NO | 0 | M | 0 | 0 | Celeston, MgSO4, PanG, Heparin |  | marginal placenta previa |  | X | X | X |
| PTL11 | 26 | 33+0 | 33+0 | 1 | 0 | 0 | Vag | NO | MC/DA | M/M | 0 | 0 | Celeston, PenG,Gravol,morfin, DICLOFENAC, anti-inflamatory drug | ' | GBS+ | African american | X |  |  |
| PTL12 | 39 | 29+6 | 29+6 | 2 | 1 | 1 | c-sec | NO | DC/DA | M/M | 0 | 1 | Celeston,MgSO4, Flagyl |  | Trichomonas + | African american | X |  |  |
| PTL13 | 34 | 28+2 | 28+2 | 1 | 0 | 0 | Vag | NO | 0 | M | 0 | 0 | PenG, Gravol, morfin, MgSO4, Celeston | Celeston at 25 week first | Cerclage at 20wks. | African american | X | X | X |
| PTL14 | 27 | 27+3 | 27+3 | 1 | 0 | 0 | c-sec | NO | DC/DA | F/F | 0 | 0 | MgSO4 | 4 and 5 Dec/14 | PCOS, hypercholesterolemia, benign breast mass-2011, GDM | Asian | X |  |  |
| PTL15 | 29 | 29+4 | 29+4 | 6 | 0 | 0 | Vag | NO | 0 | M | 0 | 0 | MgSO4, PenG,Gravol, DICLOFENAC, anti-inflamatory drug | Celeston at 27 weeks |  | Caucasian | X | X | X |
| PTL16 | 35 | 28+5 | 28+6 | 2 | 0 | 0 | Vag | NO | 0 | F | AROM | 0 | Celeston-1 dose only, MgSo4 |  | Syphilus-Aug,2014 . Chronic migrain, hyperten since age 15 | African american | X | X | X |
| PTL17 |  | 27+5 | 27+5 | 4 | 2 | 0 | Vag | NO | 0 | M | 1 | 0 | Celeston-1 dose only, MgSo4, PenG, |  | 2 normal pregn.before, GBS+, umbilical hernia repair | Caucasian | X | X | X |
| PTL18 | 26 | 23+2 | 23+2 | 1 | 0 | 0 | Vag | NO | 0 | F | 1 | 0 | Clindomycin, DICLOFENAC,Nalaxon, Tylenol, anti-inflamatory drug | Hydrocortison | Home-PROGESTERON, cerclage on Feb16/15, anhydromniosis, stillborn baby | Caucasian | X | X | X |
| PTL19 | 22 | 29+0 | 29+0 | 1 | 0 | 0 | Vag | NO | 0 | M | 0 | 0 | MgSO4, PenG, Gravol,DICLOFENAC,Tobramycine for 24h,anti-inflamatory drug | 2x bethametason, fentanyl |  | Caucasian | X | X | X |
| PTL20 | 31 | 28+0 | 28+0 | 2 | 1 | 0 | Vag | NO | 0 | M | 1 | 0 | MgSO4, PenG, Gravol, DICLOFENAC,Tylenol, NIFEdipine, anti-inflamatory drug |  | Home-PROGESTERON | Caucasian | X | X | X |

**Supporting Table 2**. List of the top 20 most abundant miRNA (miRNAs are listed in order of abundance) identified in the different sample types independent of PTL.

| **Plasma*** | **EV*** | **EV-depleted plasma*** |
| --- | --- | --- |
| **hsa-miR-451a-5p** | **hsa-miR-451a-5p** | **hsa-miR-451a-5p** |
| **hsa-miR-223-3p** | **hsa-miR-21-5p** | **hsa-miR-486-1-5p** |
| **hsa-miR-16-1-5p** | **hsa-miR-223-3p** | **hsa-miR-21-5p** |
| **hsa-miR-21-5p** | hsa-miR-26a-1-5p | **hsa-miR-92a-1-3p** |
| **hsa-miR-23a-3p** | hsa-miR-126-3p | **hsa-miR-24-1-3p** |
| **hsa-miR-486-1-5p** | **hsa-miR-16-1-5p** | hsa-miR-22-3p |
| **hsa-miR-92a-1-3p** | **hsa-miR-23a-3p** | **hsa-miR-23a-3p** |
| **hsa-miR-24-1-3p** | **hsa-miR-92a-1-3p** | **hsa-miR-16-1-5p** |
| hsa-miR-22-3p | **hsa-miR-142-5p** | hsa-miR-423-5p |
| hsa-miR-126-3p | hsa-miR-126-5p | hsa-miR-320a-3p |
| hsa-miR-19b-1-3p | hsa-miR-26b-5p | hsa-miR-19b-1-3p |
| **hsa-miR-199a-1-3p** | hsa-let-7a-1-5p | hsa-miR-146a-5p |
| hsa-miR-126-5p | hsa-miR-142-3p | **hsa-miR-142-5p** |
| **hsa-miR-142-5p** | hsa-let-7f-1-5p | **hsa-miR-223-3p** |
| hsa-miR-25-3p | **hsa-miR-486-1-5p** | **hsa-miR-199a-1-3p** |
| hsa-miR-423-5p | hsa-miR-191-5p | hsa-miR-335-5p |
| hsa-miR-27a-3p | **hsa-miR-24-1-3p** | hsa-miR-130a-3p |
| hsa-miR-30e-5p | hsa-let-7g-5p | hsa-miR-221-3p |
| hsa-miR-221-3p | hsa-miR-103a-1-3p | hsa-miR-484-5p |
| hsa-miR-30d-5p | **hsa-miR-199a-1-3p** | hsa-miR-25-3p |

**: The miRNA observed in all three different sample types are listed in boldface characters and the unique miRNA in specific sample types are underlined.*

**Supporting Table 3.** List of miRNA showing concentration difference between EV and EV-depleted fractions.

| miRNA ID | Fold change (log2) EV/EV-dep | p-value | miRNA ID | Fold change  (log2)  EV/EV-dep | p-value | miRNA ID | Fold change  (log2)  EV/EV-dep | p-value | miRNA ID | Fold change (log2)  EV/EV-dep | p-value |
| --- | --- | --- | --- | --- | --- | --- | --- | --- | --- | --- | --- |
| miR-204-3p | -1.00 | 1.53E-05 | miR-18b-3p | -1.47 | 8.01E-04 | miR-500a-3p | -2.22 | 3.71E-08 | miR-144-5p | 2.43 | 2.30E-06 |
| miR-499a-5p | -1.00 | 1.34E-03 | miR-145-5p | -1.48 | 9.42E-05 | miR-877-5p | -2.23 | 1.92E-10 | miR-590-3p | 2.42 | 2.68E-06 |
| miR-1294-5p | -1.00 | 1.21E-05 | miR-6891-5p | -1.48 | 9.62E-08 | miR-361-5p | -2.24 | 2.51E-08 | miR-135a-1-5p | 2.41 | 2.82E-09 |
| miR-6813-5p | -1.01 | 1.70E-04 | miR-7976-5p | -1.49 | 2.27E-05 | miR-3200-3p | -2.25 | 8.77E-05 | miR-195-5p | 2.40 | 1.65E-10 |
| miR-6513-3p | -1.01 | 1.91E-03 | miR-519d-5p | -1.49 | 6.01E-05 | miR-1307-5p | -2.26 | 2.24E-08 | miR-24-1-5p | 2.37 | 3.90E-11 |
| miR-376c-3p | -1.01 | 4.10E-02 | miR-378c-5p | -1.49 | 7.29E-08 | miR-215-5p | -2.27 | 7.53E-04 | miR-30b-5p | 2.36 | 8.36E-07 |
| miR-942-5p | -1.02 | 4.44E-05 | miR-532-5p | -1.49 | 1.72E-06 | miR-874-3p | -2.27 | 2.85E-08 | miR-338-3p | 2.35 | 8.54E-10 |
| miR-515-1-5p | -1.02 | 9.43E-03 | miR-1284-5p | -1.50 | 4.98E-07 | miR-1283-1-5p | -2.33 | 4.51E-06 | miR-625-5p | 2.35 | 4.38E-07 |
| miR-574-5p | -1.03 | 2.23E-04 | miR-338-5p | -1.51 | 1.34E-06 | miR-6087-5p | -2.34 | 1.74E-07 | miR-335-3p | 2.31 | 6.35E-07 |
| miR-548e-3p | -1.04 | 7.54E-04 | miR-363-3p | -1.51 | 7.55E-06 | miR-154-5p | -2.34 | 8.39E-05 | miR-30c-1-5p | 2.27 | 3.02E-05 |
| miR-4466-5p | -1.04 | 5.88E-04 | miR-125a-3p | -1.51 | 1.22E-06 | miR-452-3p | -2.36 | 7.97E-07 | miR-186-5p | 2.21 | 1.49E-09 |
| miR-517a-3p | -1.04 | 8.44E-04 | miR-520a-5p | -1.52 | 2.03E-04 | miR-518e-5p | -2.39 | 2.56E-05 | miR-204-5p | 2.20 | 3.03E-07 |
| miR-548o-3p | -1.04 | 9.57E-04 | miR-450b-5p | -1.52 | 1.25E-05 | miR-4508-5p | -2.41 | 6.10E-08 | miR-200c-3p | 2.17 | 5.04E-08 |
| miR-3909-3p | -1.04 | 1.39E-03 | miR-3124-5p | -1.52 | 1.84E-09 | miR-382-5p | -2.42 | 1.81E-06 | let-7g-5p | 2.15 | 1.38E-08 |
| miR-129-1-5p | -1.04 | 1.19E-05 | miR-1307-3p | -1.52 | 3.74E-05 | miR-589-5p | -2.44 | 7.57E-11 | miR-301a-3p | 2.15 | 2.19E-07 |
| miR-5189-5p | -1.05 | 1.07E-04 | miR-148a-5p | -1.53 | 2.47E-06 | miR-188-5p | -2.44 | 7.30E-10 | miR-518b-3p | 2.13 | 1.04E-03 |
| miR-421-3p | -1.05 | 1.54E-04 | miR-18a-3p | -1.53 | 8.88E-03 | miR-525-5p | -2.46 | 1.11E-08 | let-7d-5p | 2.09 | 7.43E-07 |
| miR-6741-5p | -1.05 | 8.95E-05 | miR-551a-3p | -1.53 | 6.01E-03 | miR-3138-3p | -2.48 | 6.04E-10 | miR-411-5p | 2.09 | 1.32E-04 |
| miR-652-3p | -1.07 | 1.22E-05 | miR-19b-1-3p | -1.54 | 2.15E-03 | miR-660-5p | -2.50 | 2.64E-10 | miR-139-5p | 2.07 | 1.33E-07 |
| miR-5189-3p | -1.07 | 3.85E-03 | miR-518b-5p | -1.54 | 4.28E-06 | miR-4732-5p | -2.50 | 1.20E-06 | miR-548c-5p | 2.06 | 2.13E-02 |
| miR-548l-5p | -1.07 | 3.99E-04 | miR-29c-3p | -1.54 | 2.44E-05 | miR-502-3p | -2.50 | 6.09E-10 | miR-125a-5p | 2.06 | 1.01E-08 |
| let-7a-1-3p | -1.09 | 2.41E-02 | miR-524-3p | -1.55 | 6.69E-05 | miR-24-1-3p | -2.52 | 5.26E-10 | miR-556-5p | 2.05 | 9.80E-09 |
| miR-1250-5p | -1.09 | 2.70E-04 | miR-324-3p | -1.55 | 2.81E-05 | miR-410-3p | -2.53 | 2.30E-06 | miR-95-3p | 2.05 | 1.83E-05 |
| miR-4738-3p | -1.09 | 2.62E-06 | miR-382-3p | -1.56 | 6.60E-04 | miR-320b-1-3p | -2.55 | 1.71E-09 | miR-518c-3p | 2.04 | 6.59E-04 |
| miR-3140-3p | -1.10 | 3.10E-05 | miR-665-3p | -1.56 | 2.58E-03 | miR-1180-3p | -2.59 | 1.57E-07 | miR-450a-1-5p | 1.98 | 9.25E-07 |
| miR-7706-3p | -1.10 | 1.52E-04 | miR-15a-5p | -1.57 | 1.95E-04 | miR-331-5p | -2.63 | 9.88E-10 | miR-151a-5p | 1.95 | 5.81E-08 |
| miR-517c-3p | -1.10 | 7.60E-04 | miR-221-3p | -1.57 | 1.42E-04 | miR-520b-5p | -2.64 | 1.97E-07 | miR-101-2-3p | 1.95 | 6.78E-07 |
| miR-486-2-5p | -1.10 | 2.32E-03 | miR-3605-3p | -1.59 | 4.54E-07 | miR-378a-3p | -2.64 | 7.83E-10 | miR-485-3p | 1.94 | 5.92E-05 |
| miR-6764-5p | -1.11 | 1.24E-05 | miR-550a-1-5p | -1.60 | 1.41E-06 | miR-335-5p | -2.65 | 1.55E-09 | miR-199b-5p | 1.91 | 7.54E-06 |
| miR-296-5p | -1.11 | 1.30E-02 | miR-431-5p | -1.61 | 1.98E-04 | miR-512-1-3p | -2.66 | 9.44E-10 | miR-181b-2-5p | 1.82 | 3.51E-06 |
| miR-29a-3p | -1.12 | 1.84E-05 | miR-320d-1-3p | -1.62 | 4.59E-05 | miR-337-5p | -2.67 | 4.30E-06 | miR-1185-1-5p | 1.78 | 2.52E-05 |
| miR-579-3p | -1.12 | 2.29E-04 | miR-92b-3p | -1.62 | 6.40E-07 | miR-1260b-5p | -2.69 | 2.63E-05 | miR-539-5p | 1.77 | 9.32E-08 |
| miR-4470-3p | -1.12 | 4.60E-07 | miR-193b-5p | -1.63 | 1.03E-09 | miR-345-5p | -2.76 | 1.00E-12 | miR-18a-5p | 1.76 | 1.66E-04 |
| miR-4454-5p | -1.12 | 2.64E-04 | miR-598-3p | -1.63 | 1.88E-06 | miR-323a-3p | -2.77 | 3.01E-07 | miR-520g-3p | 1.76 | 4.72E-03 |
| miR-1292-5p | -1.12 | 6.20E-04 | miR-656-3p | -1.64 | 7.98E-03 | miR-130a-3p | -2.86 | 1.22E-08 | miR-342-3p | 1.75 | 1.22E-06 |
| miR-636-3p | -1.13 | 4.15E-05 | miR-15b-3p | -1.65 | 7.52E-04 | miR-3615-3p | -2.87 | 2.60E-12 | miR-519d-3p | 1.73 | 1.99E-03 |
| miR-3158-1-5p | -1.13 | 8.61E-05 | miR-664a-5p | -1.66 | 2.13E-07 | miR-584-5p | -2.88 | 2.72E-09 | miR-518f-3p | 1.72 | 1.90E-04 |
| miR-520f-5p | -1.13 | 1.28E-03 | miR-124-1-3p | -1.66 | 1.75E-04 | miR-629-5p | -2.94 | 2.02E-13 | miR-27a-5p | 1.69 | 2.68E-06 |
| miR-4755-5p | -1.13 | 1.07E-06 | miR-23b-5p | -1.66 | 1.42E-04 | miR-22-3p | -2.95 | 1.12E-12 | miR-331-3p | 1.68 | 3.24E-07 |
| miR-486-1-3p | -1.14 | 1.39E-03 | miR-3605-5p | -1.67 | 3.94E-08 | miR-486-1-5p | -2.96 | 1.25E-07 | miR-153-1-3p | 1.65 | 1.78E-06 |
| miR-485-5p | -1.15 | 2.24E-02 | miR-6852-5p | -1.67 | 1.57E-05 | miR-4286-5p | -2.96 | 4.83E-09 | miR-758-3p | 1.64 | 3.40E-04 |
| miR-3176-3p | -1.16 | 3.90E-05 | let-7f-1-3p | -1.67 | 2.61E-06 | miR-433-3p | -3.00 | 6.29E-07 | miR-27b-5p | 1.54 | 1.80E-06 |
| miR-376a-1-3p | -1.16 | 4.97E-02 | miR-5581-3p | -1.67 | 1.07E-08 | miR-193a-5p | -3.05 | 1.65E-11 | miR-4485-3p | 1.51 | 1.23E-02 |
| miR-132-3p | -1.19 | 1.98E-05 | miR-191-3p | -1.68 | 1.22E-06 | miR-515-1-3p | -3.06 | 2.94E-11 | miR-26a-1-3p | 1.50 | 1.26E-05 |
| miR-1304-5p | -1.19 | 5.04E-04 | miR-92a-1-3p | -1.70 | 5.06E-06 | miR-423-5p | -3.08 | 1.25E-10 | miR-409-5p | 1.49 | 1.09E-05 |
| miR-625-3p | -1.19 | 2.29E-02 | miR-4785-3p | -1.73 | 3.79E-07 | miR-210-3p | -3.08 | 2.73E-11 | miR-7641-1-3p | 1.47 | 4.97E-05 |
| miR-1976-5p | -1.20 | 1.62E-05 | miR-3177-3p | -1.75 | 2.02E-07 | miR-501-3p | -3.14 | 1.48E-10 | miR-340-5p | 1.47 | 3.69E-05 |
| miR-3940-3p | -1.21 | 2.90E-04 | miR-490-5p | -1.75 | 2.30E-03 | miR-627-5p | -3.17 | 3.66E-12 | miR-17-5p | 1.47 | 3.79E-04 |
| miR-1468-5p | -1.21 | 6.34E-07 | miR-548q-5p | -1.76 | 2.41E-05 | miR-130b-3p | -3.22 | 2.55E-11 | miR-487a-3p | 1.47 | 2.41E-03 |
| miR-299-3p | -1.22 | 1.32E-02 | miR-140-3p | -1.77 | 4.04E-07 | miR-1246-5p | -3.28 | 3.79E-06 | miR-29b-1-3p | 1.43 | 4.30E-03 |
| miR-7854-3p | -1.22 | 8.04E-03 | miR-362-3p | -1.78 | 9.72E-09 | miR-483-5p | -3.29 | 2.67E-11 | miR-361-3p | 1.42 | 1.04E-06 |
| miR-512-1-5p | -1.22 | 1.00E-04 | miR-1228-5p | -1.80 | 1.73E-08 | miR-519a-1-5p | -3.35 | 3.83E-07 | miR-30b-3p | 1.42 | 4.50E-07 |
| miR-2277-3p | -1.22 | 1.49E-04 | miR-1285-1-3p | -1.80 | 4.39E-06 | miR-524-5p | -3.38 | 1.08E-09 | miR-4485-5p | 1.39 | 9.97E-03 |
| miR-6809-5p | -1.24 | 9.50E-07 | miR-1287-5p | -1.83 | 1.51E-07 | miR-134-5p | -3.51 | 7.05E-09 | miR-1277-5p | 1.39 | 8.85E-04 |
| miR-551b-3p | -1.24 | 5.92E-04 | miR-520a-3p | -1.84 | 3.97E-07 | miR-323b-3p | -3.54 | 8.20E-08 | miR-136-5p | 1.39 | 3.44E-05 |
| miR-2110-5p | -1.25 | 1.87E-04 | miR-369-3p | -1.84 | 3.06E-04 | miR-375-3p | -3.82 | 4.90E-06 | miR-219a-1-5p | 1.38 | 1.26E-04 |
| miR-518c-5p | -1.25 | 5.89E-05 | miR-424-5p | -1.85 | 1.31E-06 | miR-320a-3p | -3.98 | 2.35E-13 | miR-24-2-5p | 1.37 | 7.92E-05 |
| let-7f-2-3p | -1.26 | 1.03E-05 | miR-122-5p | -1.85 | 2.99E-02 | miR-4532-5p | -4.71 | 3.30E-09 | miR-381-3p | 1.35 | 4.14E-03 |
| miR-605-5p | -1.26 | 2.60E-03 | miR-550a-3-5p | -1.86 | 6.83E-05 | miR-142-3p | 4.52 | 6.49E-14 | miR-223-3p | 1.34 | 1.14E-03 |
| miR-4676-3p | -1.27 | 2.51E-03 | miR-760-3p | -1.88 | 1.53E-06 | let-7f-2-5p | 4.35 | 5.41E-15 | miR-494-3p | 1.33 | 7.20E-04 |
| miR-660-3p | -1.27 | 3.15E-04 | miR-2276-3p | -1.89 | 4.07E-08 | miR-374a-5p | 4.30 | 2.71E-14 | miR-103a-1-3p | 1.32 | 2.94E-04 |
| miR-32-5p | -1.27 | 2.20E-03 | miR-21-3p | -1.91 | 3.54E-07 | miR-454-3p | 4.08 | 5.63E-13 | miR-30a-5p | 1.32 | 5.11E-06 |
| miR-10a-3p | -1.28 | 4.17E-06 | miR-145-3p | -1.91 | 1.03E-07 | miR-1277-3p | 3.95 | 1.58E-10 | miR-641-5p | 1.30 | 1.82E-04 |
| miR-543-5p | -1.28 | 3.27E-03 | miR-148b-3p | -1.92 | 1.63E-08 | miR-374b-5p | 3.91 | 1.79E-11 | miR-32-3p | 1.25 | 3.27E-03 |
| miR-550a-1-3p | -1.28 | 8.39E-03 | miR-100-5p | -1.92 | 2.06E-09 | miR-340-3p | 3.89 | 1.62E-11 | miR-30c-1-3p | 1.25 | 4.44E-05 |
| miR-326-3p | -1.28 | 1.70E-04 | miR-9-1-3p | -1.93 | 1.78E-09 | let-7f-1-5p | 3.79 | 1.70E-14 | miR-151b-3p | 1.25 | 1.68E-04 |
| miR-452-5p | -1.28 | 5.09E-03 | miR-942-3p | -1.95 | 1.07E-11 | miR-26a-1-5p | 3.76 | 5.54E-14 | miR-106a-5p | 1.24 | 4.12E-03 |
| miR-590-5p | -1.29 | 1.01E-02 | let-7g-3p | -1.95 | 2.78E-06 | miR-107-3p | 3.75 | 7.64E-12 | miR-6516-5p | 1.24 | 1.14E-04 |
| miR-380-3p | -1.29 | 7.67E-03 | miR-4732-3p | -1.96 | 5.28E-05 | miR-26b-5p | 3.66 | 2.15E-13 | miR-487a-5p | 1.24 | 2.84E-07 |
| miR-323a-5p | -1.30 | 7.00E-04 | miR-654-3p | -1.98 | 9.63E-05 | miR-98-5p | 3.55 | 3.00E-12 | miR-1256-5p | 1.23 | 1.79E-04 |
| miR-2116-3p | -1.32 | 2.13E-05 | miR-511-5p | -2.01 | 1.68E-09 | let-7e-5p | 3.36 | 9.59E-11 | miR-362-5p | 1.22 | 1.69E-05 |
| miR-629-3p | -1.32 | 3.90E-06 | miR-934-3p | -2.02 | 3.45E-07 | miR-190a-5p | 3.35 | 3.95E-11 | miR-618-5p | 1.22 | 1.97E-02 |
| miR-329-1-3p | -1.32 | 2.07E-03 | miR-152-3p | -2.04 | 8.69E-08 | let-7a-1-5p | 3.30 | 1.13E-13 | miR-301b-3p | 1.21 | 4.17E-04 |
| miR-19a-3p | -1.33 | 1.18E-02 | let-7d-3p | -2.05 | 4.92E-06 | miR-155-5p | 3.23 | 2.37E-12 | miR-103a-2-3p | 1.20 | 2.13E-05 |
| mir-1273a | -1.34 | 3.33E-04 | miR-484-5p | -2.05 | 1.91E-07 | miR-150-5p | 3.01 | 3.32E-09 | miR-20a-5p | 1.19 | 6.69E-03 |
| miR-4714-3p | -1.34 | 1.77E-07 | miR-520d-5p | -2.06 | 8.83E-06 | miR-374b-3p | 2.96 | 1.24E-11 | miR-30a-3p | 1.19 | 1.49E-04 |
| miR-889-3p | -1.34 | 1.86E-03 | miR-4433b-3p | -2.07 | 3.16E-08 | miR-20a-3p | 2.95 | 5.88E-10 | miR-26a-2-3p | 1.19 | 1.76E-04 |
| miR-339-5p | -1.35 | 5.40E-03 | miR-518a-1-5p | -2.08 | 1.56E-06 | miR-181c-5p | 2.94 | 2.84E-10 | miR-491-5p | 1.19 | 2.59E-05 |
| miR-96-5p | -1.36 | 3.50E-02 | miR-2355-3p | -2.08 | 1.29E-07 | miR-126-3p | 2.89 | 1.43E-11 | miR-29a-5p | 1.16 | 7.50E-05 |
| miR-1228-3p | -1.37 | 3.14E-05 | miR-3120-3p | -2.10 | 2.77E-07 | miR-374a-3p | 2.84 | 5.46E-08 | miR-505-5p | 1.16 | 7.09E-06 |
| miR-1306-5p | -1.38 | 4.46E-03 | miR-22-5p | -2.10 | 1.90E-05 | miR-199a-1-5p | 2.82 | 9.78E-10 | miR-324-5p | 1.16 | 2.28E-05 |
| miR-3143-5p | -1.38 | 5.24E-04 | miR-6791-3p | -2.10 | 2.05E-08 | miR-146b-5p | 2.76 | 3.13E-10 | miR-6087-3p | 1.15 | 1.88E-04 |
| miR-498-5p | -1.39 | 5.15E-04 | miR-136-3p | -2.11 | 5.51E-05 | miR-29b-2-3p | 2.71 | 2.73E-10 | miR-191-5p | 1.15 | 6.90E-05 |
| miR-148a-3p | -1.40 | 8.28E-07 | miR-885-5p | -2.11 | 2.69E-03 | miR-28-5p | 2.69 | 1.29E-09 | miR-7-1-5p | 1.15 | 2.41E-04 |
| miR-192-5p | -1.40 | 6.11E-04 | miR-3187-3p | -2.11 | 4.33E-10 | miR-30e-3p | 2.65 | 1.28E-10 | miR-29c-5p | 1.14 | 2.74E-03 |
| miR-183-3p | -1.40 | 3.47E-03 | miR-4710-5p | -2.12 | 4.03E-07 | miR-23b-3p | 2.64 | 4.56E-11 | miR-545-5p | 1.12 | 3.86E-04 |
| miR-451a-3p | -1.41 | 3.05E-03 | miR-339-3p | -2.12 | 8.84E-09 | let-7c-5p | 2.63 | 5.46E-10 | miR-18b-5p | 1.12 | 8.83E-03 |
| miR-431-3p | -1.42 | 4.91E-04 | miR-3667-5p | -2.13 | 2.15E-05 | miR-628-5p | 2.58 | 3.19E-08 | miR-127-3p | 1.11 | 3.58E-04 |
| miR-4701-5p | -1.42 | 1.17E-05 | miR-4685-3p | -2.14 | 6.06E-07 | miR-19b-2-3p | 2.55 | 2.85E-12 | miR-941-1-3p | 1.10 | 9.86E-04 |
| miR-193b-3p | -1.42 | 2.10E-05 | miR-23a-5p | -2.15 | 4.23E-07 | miR-194-2-5p | 2.54 | 1.84E-05 | miR-493-3p | 1.09 | 1.24E-02 |
| miR-3656-3p | -1.43 | 1.06E-05 | miR-3679-5p | -2.15 | 3.86E-08 | let-7a-3-5p | 2.53 | 2.77E-09 | miR-20b-5p | 1.09 | 2.36E-02 |
| miR-6877-5p | -1.44 | 3.58E-04 | miR-146a-5p | -2.18 | 1.40E-07 | miR-148b-5p | 2.51 | 3.18E-08 | miR-31-5p | 1.08 | 1.79E-04 |
| miR-423-3p | -1.44 | 2.14E-05 | let-7b-3p | -2.19 | 5.41E-06 | miR-664a-3p | 2.49 | 5.19E-09 | miR-379-5p | 1.07 | 1.38E-02 |
| miR-532-3p | -1.45 | 1.35E-05 | miR-205-5p | -2.19 | 7.97E-07 | miR-221-5p | 2.45 | 5.96E-09 | miR-203a-3p | 1.06 | 3.58E-02 |
| miR-424-3p | -1.45 | 1.30E-06 | miR-576-3p | -2.19 | 2.21E-09 | miR-26a-2-5p | 2.44 | 1.26E-08 | miR-299-5p | 1.06 | 5.62E-03 |
| miR-6515-5p | -1.45 | 4.31E-08 | miR-4659b-5p | -2.20 | 5.91E-10 | let-7a-2-5p | 2.44 | 3.47E-11 | miR-493-5p | 1.04 | 1.30E-02 |
| miR-1908-5p | -1.46 | 2.89E-06 | miR-526b-5p | -2.21 | 5.47E-07 | miR-1249-3p | 2.43 | 2.08E-09 | miR-548ax-5p | 1.02 | 1.06E-04 |
| miR-99a-5p | -1.46 | 1.94E-06 |  |  |  |  |  |  |  |  |  |

**Supporting Table 4.**  List of other circulating RNAs affected by PTL

| **Category** | **Match ID** | **Fold change (PTL/Control, log2)** | | | **p-value** | | |
| --- | --- | --- | --- | --- | --- | --- | --- |
|  |  | Plasma | EV | EV-dep Plasma | Plasma | EV | EV-dep Plasma |
| **LncRNA** | lnc-TRIM69-2:2 |  | **1.96** | **1.64** |  | 2.55E-03 | 2.30E-03 |
|  | lnc-AC087650.1-1:1 | **1.83** |  | **1.01** | 8.04E-06 |  | 1.32E-02 |
|  | lnc-NRGN-2:1 | **1.01** | **1.53** |  | 4.32E-02 | 3.99E-02 |  |
|  | lnc-ACTB-2:2 |  | **1.17** |  |  | 1.55E-04 |  |
|  | lnc-POTEH-1:1 |  | **1.22** |  |  | 3.89E-03 |  |
|  | lnc-AC013269.5.1-3:1 |  | **1.02** |  |  | 3.34E-03 |  |
|  | lnc-PDIA4-1:1 |  | **1.34** |  |  | 7.55E-03 |  |
| **SnoRNA** | Snord26 | **1.76** |  | **1.42** | 7.37E-05 |  | 2.78E-02 |
|  | snord88A | **1.30** |  | **1.04** | 6.01E-05 |  | 2.11E-02 |
|  | snord14A | **1.00** |  | **1.01** | 1.58E-03 |  | 2.83E-03 |
|  | snord95 | **1.40** |  |  | 2.98E-05 |  |  |
|  | Snord22 | **1.62** |  |  | 1.33E-04 |  |  |
|  | snord20 | **1.01** |  |  | 3.90E-03 |  |  |
|  | snord33 | **1.01** |  |  | 2.21E-03 |  |  |
|  | snora77 | **1.48** |  |  | 5.02E-05 |  |  |
|  | Snord31 | **1.08** |  |  | 1.22E-02 |  |  |
|  | snou2-19 | **1.02** |  |  | 5.94E-05 |  |  |
| **piRNA** | piR-hsa-12485 |  | **1.13** | **1.03** |  | 6.11E-03 | 2.93E-03 |
|  | piR-hsa-27140 | **1.76** |  | **1.51** | 2.38E-05 |  | 2.83E-04 |
|  | piR-hsa-26872 | **1.78** |  | **1.23** | 1.73E-07 |  | 1.60E-03 |
|  | piR-hsa-20613 |  |  | **1.24** |  |  | 5.78E-03 |
|  | piR-hsa-26592 |  |  | **2.48** |  |  | 4.04E-02 |
|  | piR-hsa-28382 |  | **-1.25** |  |  | 6.44E-03 |  |
|  | piR-hsa-31238 |  | **-1.81** |  |  | 1.90E-02 |  |
|  | piR-hsa-31237 |  | **1.33** |  |  | 3.48E-02 |  |
|  | piR-hsa-28593 | **1.35** |  |  | 2.96E-04 |  |  |
|  | piR-hsa-24684 | **1.17** |  |  | 9.39E-03 |  |  |
|  | piR-hsa-30715 | **1.06** |  |  | 1.82E-03 |  |  |

**References**

**1. Baranyai T, Herczeg K, Onodi Z, *et al*.** Isolation of Exosomes from Blood Plasma: Qualitative and Quantitative Comparison of Ultracentrifugation and Size Exclusion Chromatography Methods. *PLoS ONE*. 2015. DOI: 10.1371/journal.pone.0145686
